# Supplementary material for: Dynamic breaking of a single gold bond
Source: Nat Commun. 2017 Jul 17;8:15931. doi: 10.1038/ncomms15931 (PMC5520017; doi:10.1038/ncomms15931)
Supplement: Supplementary Information [file ncomms15931-s1.pdf]

Type of file: PDF
Size of file: 0 KB
Title of file for HTML: Supplementary Information
Description: Supplementary Figures, Supplementary Notes, Supplementary Tables and Supplementary References

Type of file: PDF
Size of file: 0 KB
Title of file for HTML: Peer Review File
Description:

Supplementary Note 1: The rate theory model of the bond breaking process

In this section we formulate the rate theory model of the bond breaking during stretching that was employed to analyse simulation results (see main text).

1.1 Model assumptions

Rate theory considers a reaction as a one-dimensional energy landscape with a potential minimum, corresponding to the equilibrium bound state, and separated from the unbound state by a potential barrier. The model treats the bond-breaking process as a kinetic problem of the thermally-activated escape from the bound state by means of the barrier-crossing. It is described as a first-order process with the dissociation rate

$$r(t) = -\frac{dP}{dt} = RP(t) \tag{S1}$$

where $P(t)$ is the occupation function describing population of the bound state and R is the rate constant given by the Arrhenius-type expression

$$R = \omega_a \exp\left(-\frac{E_a}{k_B T}\right) \tag{S2}$$

where ω_a is the attempt frequency, T is the absolute temperature and k_B is the Boltzmann constant. Before stretching (the stretching length $l = l_0$ or extension $\Delta l = l - l_0 = 0$) the bound and the unbound states are separated by an energy barrier $E_a(l_0)$, and the rate constant is

$$R(l_0) \equiv R_0 = \omega_a \exp\left(-\frac{E_a(l_0)}{k_B T}\right) \tag{S3}$$

The application of force F in the direction of the barrier tilts the energy landscape and decreases the energy barrier, thus facilitating escape. Further we consider three linear approximations: the extension Δl increases in time linearly with the probe stretching rate v,

$$\Delta l = l - l_0 = vt \tag{S4}$$

the loading force increases as a linear function of l with an effective spring constant k_s;

$$F = F_0 + k_s \Delta l \tag{S5}$$

and the energy barrier decreases as a linear function of stretching distance l with the slope α_l:

$$E_a(l) = E_a(l_0) - \alpha_l \Delta l \tag{S6}$$

As the parameters ω_a, $E_a(l_0)$, α_l are determined by the shape of the reaction landscape, we assume them to be temperature-independent. Combining equations S2-S6, one obtains for the rate constant of dissociation during the stretching

$$R = \omega_a \exp\left(-\frac{E_a(l_0) - \alpha_l \Delta l}{k_B T}\right) = \omega_a \exp\left(-\frac{E_a(l_0)}{k_B T}\right) \exp\left(\frac{\alpha_l \Delta l}{k_B T}\right) = R_0 \exp\left(\frac{\alpha_l vt}{k_B T}\right) \tag{S7}$$

1.2 Occupation function

By inserting Equations S7 into Equation S1, one obtains

$$\frac{dP}{dt} = -R_0 \exp\left(\frac{\alpha_l v t}{k_B T}\right) P(t) \tag{S8}$$

The solution of Equation S8 with the limiting conditions $P(t=0) = 1$ and $P(t \to \infty) = 0$ is given by

$$P(t) = \exp\left[\frac{k_B T R_0}{\alpha_l v}\left(1 - \exp\left(\frac{\alpha_l v t}{k_B T}\right)\right)\right] \tag{S9}$$

Here we can define the rate constant of mechanical impact

$$R_M = \frac{\alpha_l v}{k_B T} \tag{S10}$$

and rewrite Equation S9 as

$$P(t) = \exp\left[\frac{R_0}{R_M}\left(1 - \exp\left(R_M t\right)\right)\right] \tag{S11}$$

Note that for small stretching rates we obtain $R_M t \to 0$ and

$$P_0(t) = \exp\left[\frac{R_0}{R_M}\left(1 - 1 - R_M t\right)\right] = \exp\left[-R_0 t\right] \tag{S12}$$

which is the solution of equation S1 in the absence of the loading force ($R = R_0$).

To illustrate the effect of loading force on the kinetics of bond dissociation, we introduce two dimensionless variables $x = R_0 t$ and the quotient of the two characteristic rate constants Q

$$Q = \frac{R_M}{R_0} = \frac{\alpha_l v}{k_B T R_0} = \frac{v}{v_c} \tag{S13}$$

where

$$v_c = \frac{k_B T R_0}{\alpha_l} \tag{S14}$$

is the probe stretching rate at which $R_M = R_0$, further referred to as the critical value of v.

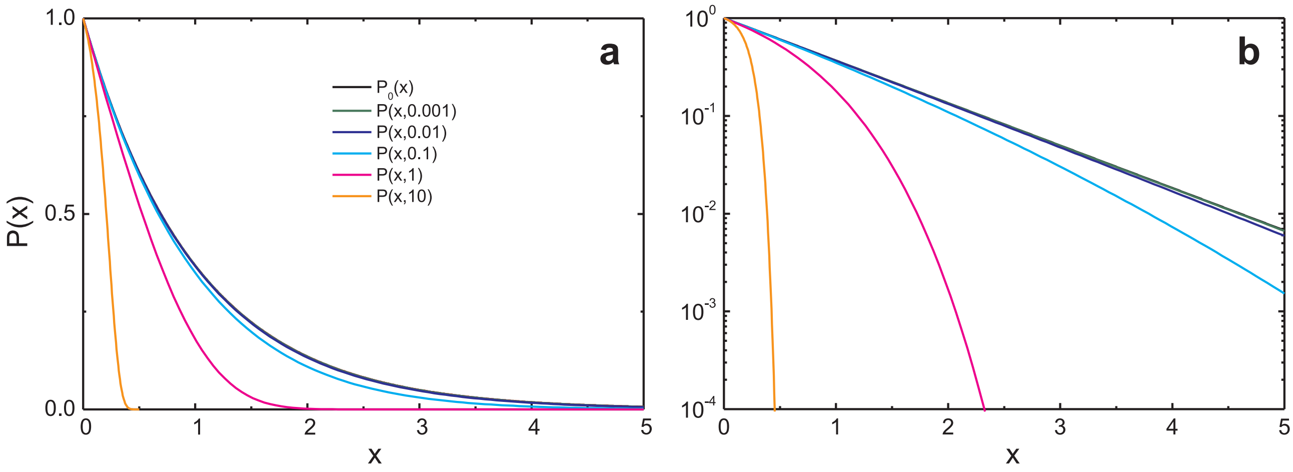

Supplementary Figure 1: Occupation curves simulated according to Equations S15 and S16 and presented in linear (a) and logarithmic (b) scale.

The occupation functions in the presence and in the absence of stretching are thus given by

$$P_0(x) = \exp(-x) \tag{S15}$$

and

$$P(x,Q) = \exp\left[\frac{1}{Q}\left(1 - \exp(Qx)\right)\right] \tag{S16}$$

The curves simulated according to Equations S15 and S16 (Supplementary Figure 1) demonstrate that $P(x,Q)$ is practically indistinguishable from $P_0(x)$ in the range of $0 < x < 5$ (corresponds to $> 99\%$ decay of P_0) up to $Q = 0.001$. At higher Q the decay of occupation function is faster.

1.3 Most probable stretching time, extension and breaking force

The rate of bond breaking $r(t)$ at the moment t is given by the derivative of the occupation function $P(t)$

$$r(t) = -\frac{dP}{dt} = R_0 \exp\left(R_M t\right) \exp\left[\frac{R_0}{R_M}\left(1 - \exp\left(R_M t\right)\right)\right] \tag{S17}$$

Importantly, $r(t)$ is also the probability density of stretching times, meaning that a higher breaking rate corresponds to a more frequently occurring stretching time. Similar to above, we introduce for the purposes of illustration a dimensionless function $r(x,Q) = r(t)/R_0$

$$r(x,Q) = \exp(Qx) \exp\left[\frac{1}{Q}\left(1 - \exp(Qx)\right)\right] \tag{S18}$$

Examples of simulated bond breaking rate curves are given in Supplementary Figure 2. They exhibit one maximum, which position t^* can be determined by solving an equation

$$\frac{dr}{dt}(t^*) = 0 \tag{S19}$$

Its solution is given by

$$t^* = \frac{1}{R_M} \ln\left(\frac{R_M}{R_0}\right) = \frac{k_B T}{\alpha_l v} \ln\left(\frac{\alpha_l v}{k_B T R_0}\right) \tag{S20}$$

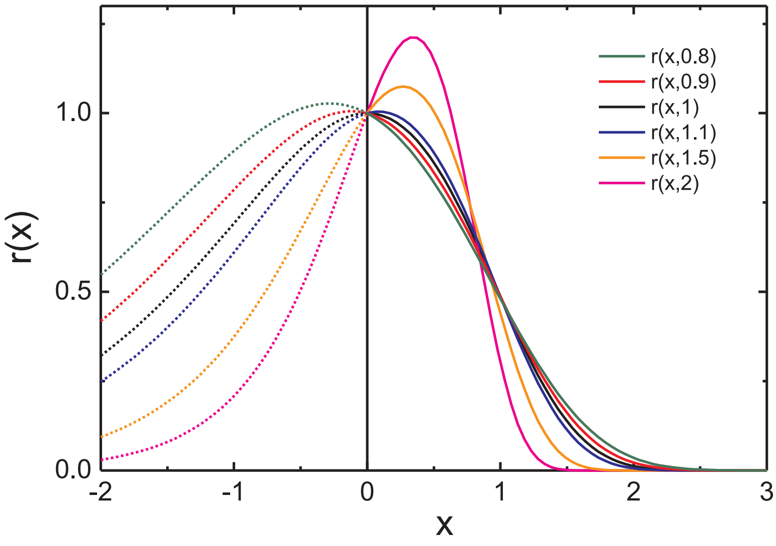

Supplementary Figure 2: Bond breaking rate as a function of dimensionless time simulated according to Equation S18 for different values of Q.

The corresponding most probable bond extension before breaking Δl^* is given by

$$\Delta l^* = \frac{k_B T}{\alpha_l} \ln\left(\frac{\alpha_l v}{k_B T R_0}\right) \tag{S21}$$

and the force increase corresponding to the maximum of detachment rate, and therefore the most probable breaking force $F^* = \Delta F^* + F_0$ is given by

$$\Delta F^* = k_s \Delta l^* = \frac{k_s k_B T}{\alpha_l} \ln\left(\frac{\alpha_l v}{k_B T R_0}\right) = \frac{k_s k_B T}{\alpha_l} \ln(v) + \frac{k_s k_B T}{\alpha_l} \ln\left(\frac{\alpha_l}{k_B T R_0}\right) \tag{S22}$$

The latter is a version of the main equation of the Bell-Evans model. As illustrated by curves in Supplementary Figure 2, if $Q < 1$, i.e. at slow stretching rates, the peak of distribution is located at $t^* < 0$. In this case Equation S22 gives a non-physical value $\Delta F^* < 0$. Ref. 1 describes this situation as following: "*Below the critical rate of loading, no strength was perceived for the bond, because the peak in the distribution of rupture forces stayed at zero force*". In other words, if one considers only physically-tangible times $t \geq 0$, the maximum of distribution is located at $\Delta F^* = 0$.

1.4 The activationless limit

As described above, the increase of the stretching distance and force with the increase of the stretching rate is caused by the decrease of the activation barrier with the stretching of the bond. Clearly, this increase is limited by the decrease of energy barrier to zero. This is also the limit of the applicability of rate theory, so we cannot explicitly use it beyond this point. We can, however, use this limit to predict when the transition to the activationless regime will occur. We now introduce the "*activationless limit*", the situation at which the most probable bond extension Δl^* corresponds to zero activation energy, and denote corresponding most probable stretching length (or extension), most probable stretching time and most probable breaking force as l_{al} (or Δl_{al}), t_{al} and F_{al}. Following this definition, the former and the latter are given by (Equations S6 and S5)

$$E_a(l_{al}) = E_a(l_0) - \alpha_l (l_{al} - l_0) = 0\,;\; \Delta l_{al} = l_{al} - l_0 = \frac{E_a(l_0)}{\alpha_l} \tag{S23}$$

$$F_{al} = F_0 + \frac{k_s E_a(l_0)}{\alpha_l} \tag{S24}$$

From Equation S21 we can find the stretching rate v_{al} corresponding to this situation:

$$\Delta l_{al} = \frac{E_a(l_0)}{\alpha_l} = \frac{k_B T}{\alpha_l} \ln\left(\frac{\alpha_l v_{al}}{k_B T R_0}\right) \tag{S25}$$

$$v_{al} = \frac{k_B T}{\alpha_l} R_0 \exp\left(\frac{E_a(l_0)}{k_B T}\right) = \frac{k_B T}{\alpha_l} \omega_a \tag{S26}$$

and the corresponding most probable stretching time t_{al}

$$t_{al} = \frac{E_a(l_0)}{\alpha_l v_{al}} = \frac{E_a(l_0)}{k_B T \omega_a} \tag{S27}$$

Within the considered model, the most probable bond stretching length and its breaking force cannot be higher than l_{al} and F_{al}. In case of a stretching rate $v > v_{al}$, the most probable extension is Δl_{al} and the most probable breaking force is F_{al}, however the time required to reach this situation will be $t = \Delta l_{al}/v < t_{al}$.

1.5 Mean stretching time, stretching length and breaking force

Besides the most probable values of stretching time, stretching length and breaking force, we also consider their mean values. As discussed above, their accessible range in the general case is restricted by the activationless limit. As $r(t)$ is the (not necessarily normalized) probability density of stretching times, the mean stretching time t_m is defined as

$$t_m = \int_0^{\Delta l_{al}/v} t\, r(t)\, dt \Big/ \int_0^{\Delta l_{al}/v} r(t)\, dt \tag{S28}$$

The denominator in Equation S28 is given by (c.f. Equations S1, S9, S23)

$$\begin{aligned}\int_0^{\Delta l_{al}/v} r(t)\, dt &= -\int_0^{\Delta l_{al}/v} \frac{dP}{dt}\, dt = P(0) - P(\Delta l_{al}/v) \\ &= 1 - \exp\left[\frac{R_0}{R_M}\left(1 - \exp\left(\frac{E_a(l_0)}{k_B T}\right)\right)\right] = 1 - \exp\left[\frac{1 - Q_{al}}{Q}\right]\end{aligned} \tag{S29}$$

Here we use the expression for $P(t)$ introduced in Equation S11 as well as the quotient Q introduced in Equation S13. Q_{al} is a new dimensionless model parameter corresponding to the value of Q for $v = v_{al}$

$$Q_{al} = \frac{v_{al}}{v_c} = \exp\left(\frac{E_a(l_0)}{k_B T}\right) \tag{S30}$$

For the purpose of further discussion we note that Q_{al} is a big number.

The numerator in Equation S28 can be calculated applying first integration by parts

$$\begin{aligned}\int_0^{\Delta l_{al}/v} t\, r(t)\, dt &= -\int_0^{\Delta l_{al}/v} t\frac{dP}{dt}\, dt = \int_0^{\Delta l_{al}/v} P\, dt - (tP(t))\Big|_0^{\Delta l/v} \\ &= \int_0^{\Delta l_{al}/v} P\, dt - \frac{\Delta l_{al}}{v}\exp\left[\frac{1 - Q_{al}}{Q}\right]\end{aligned} \tag{S31}$$

$$\begin{aligned}\int_0^{\Delta l_{al}/v} P dt &= \int_0^{\Delta l_{al}/v} \exp\left[\frac{1}{Q}(1 - \exp(R_M t))\right] dt \\ \int_0^{\Delta l_{al}/v} P dt &= \exp\left(\frac{1}{Q}\right)\int_0^{\Delta l_{al}/v} \exp\left[-\frac{\exp(R_M t)}{Q}\right] dt\end{aligned} \tag{S32}$$

then introducing a substitution g

$$g(t) = \frac{\exp(R_M t)}{Q},\ dg = \frac{R_M \exp(R_M t)}{Q} dt = R_M\, g\, dt,\ dt = \frac{dg}{R_M\, g} \tag{S33}$$

$$g(0) = \frac{1}{Q},\ g(\Delta l_{al}/v) = \frac{1}{Q}\exp\left(\frac{\alpha_l v}{k_B T}\frac{\Delta l_{al}}{v}\right) = \frac{1}{Q}\exp\left(\frac{E_a(l_0)}{k_B T}\right) = \frac{Q_{al}}{Q} \tag{S34}$$

rewriting the remaining integral in Equation S32 as

$$\int_0^{\Delta l_{al}/v} \exp\left[-\frac{\exp(R_M t)}{Q}\right] dt = \frac{1}{R_M}\int_{1/Q}^{Q_{al}/Q} \frac{\exp(-g)}{g} dg \tag{S35}$$

and splitting the last integral in Equation S35

$$\int_{1/Q}^{Q_{al}/Q} \frac{\exp(-g)}{g} dg = \int_{1/Q}^{+\infty} \frac{\exp(-g)}{g} dg - \int_{Q_{al}/Q}^{+\infty} \frac{\exp(-g)}{g} dg = E_1\left(\frac{1}{Q}\right) - E_1\left(\frac{Q_{al}}{Q}\right) \tag{S36}$$

The obtained function $E_1(x)$ is known as the exponential integral:

$$E_1(x) = \int_x^{+\infty} \frac{\exp(-g)}{g} dg \tag{S37}$$

Combining everything together, we obtain for the mean stretching time

$$t_m = \frac{\frac{1}{R_M}\exp\left(\frac{1}{Q}\right)\left[E_1\left(\frac{1}{Q}\right) - E_1\left(\frac{Q_{al}}{Q}\right)\right] - \frac{\Delta l_{al}}{v}\exp\left[\frac{1-Q_{al}}{Q}\right]}{1-\exp\left[\frac{1-Q_{al}}{Q}\right]} \tag{S38}$$

Note that

$$\frac{\Delta l_{al}}{v} = \frac{E_a(l_0)}{\alpha_l v} = \frac{E_a(l_0)}{k_B T}\cdot\frac{k_B T R_0}{\alpha_l v}\cdot\frac{1}{R_0} = \frac{\ln(Q_{al})}{Q R_0} \tag{S39}$$

We further rewrite Equation S38 in the form containing only Q, Q_{al} and a constant factor

$$t_m = \frac{1}{R_0}\cdot\frac{1}{Q}\cdot\frac{\exp\left(\frac{1}{Q}\right)\left[E_1\left(\frac{1}{Q}\right) - E_1\left(\frac{Q_{al}}{Q}\right)\right] - \ln(Q_{al})\exp\left[\frac{1-Q_{al}}{Q}\right]}{1-\exp\left[\frac{1-Q_{al}}{Q}\right]} = \frac{1}{R_0}\cdot\frac{1}{Q}\cdot I_m(Q,Q_{al}) \tag{S40}$$

The corresponding mean extension can be obtained as

$$\Delta l_m = v t_m = \frac{v}{R_0 Q}\cdot I_m(Q,Q_{al}) = \frac{k_B T}{\alpha_l} I_m(Q,Q_{al}) \tag{S41}$$

and the mean increase of breaking force can be found from its proportionality to Δl_m.

The introduced function $I_m(Q,Q_{al})$ is thus producing mean values of stretching times, stretching length and breaking force. For comparison, we rewrite expressions for the most probable values in the same form:

$$t^* = \frac{1}{R_0}\cdot\frac{1}{Q}\ln(Q) = \frac{1}{R_0}\cdot\frac{1}{Q}\cdot I^*(Q,Q_{al}),\ \Delta l^* = \frac{k_B T}{\alpha_l}\ln(Q) = \frac{k_B T}{\alpha_l} I^*(Q,Q_{al}) \tag{S42}$$

and, taking into accounts limits imposed by the critical stretching rate and the activationless limit, the function $I^*(Q,Q_{al})$ producing most probable values is given by

$$I^*(Q,Q_{al}) = \begin{cases} 0 & \text{if } Q \le 1 \\ \ln(Q) & \text{if } 1 \le Q \le Q_{al} \\ \ln(Q_{al}) & \text{if } Q \ge Q_{al} \end{cases} \tag{S43}$$

The behavior of I_m and I^*, which are proportional to mean and most probable bond extension is illustrated in Supplementary Figure 3. They both shows three characteristic regimes corresponding to spontaneous breaking, force-assisted breaking and activationless limit. In case of I_m the transition between regimes is rather smooth and resembles the curves obtained in experiments and simulations better than the abrupt transition exhibited by I^*. We note that the qualitative appearance of both I_m and I^* is not changing as long as Q_{al} is large enough.

To explore the behavior of I_m in three different regimes, we employ two different approximations for $E_1(x)$

$$E_1(x) \approx \frac{\exp(-x)}{x} \text{ for large } x \tag{S44}$$

$$E_1(x) \approx -\gamma - \ln(x) + x \text{ for small } x \tag{S45}$$

where $\gamma \approx 0.577$ is Euler-Mascheroni constant[2]. In spontaneous breaking regime $Q << 1$ and both $E_1(1/Q)$ and $E_1(Q_{al}/Q)$ can be approximated according to Equation S44, furthermore $\exp[(1-Q_{al})/Q] \to 0$. Then one obtains

$$I_m(Q) \approx \frac{Q - \frac{Q}{Q_{al}}\exp\left[\frac{1-Q_{al}}{Q}\right] - \ln(Q_{al})\exp\left[\frac{1-Q_{al}}{Q}\right]}{1-\exp\left[\frac{1-Q_{al}}{Q}\right]} \approx Q \tag{S46}$$

By definition I_m is never zero at any finite stretching rate, but the linear dependence of stretching length and breaking force on stretching rate is probably too weak to be reliably detected in experiments.

In force-assisted regime $1 << Q << Q_{al}$; $E_1(1/Q)$ is approximated according to Equation S45 and $E_1(Q_{al}/Q)$ according to Equation S44, furthermore $\exp[(1-Q_{al})/Q] \to 0$, $1/Q \to 0$ and $\exp[1/Q] \to 1$

$$I_m \approx \frac{\ln(Q) - \gamma + \dfrac{1}{Q} - \dfrac{Q}{Q_{al}} \exp\left[\dfrac{1-Q_{al}}{Q}\right] - \ln(Q_{al}) \exp\left[\dfrac{1-Q_{al}}{Q}\right]}{1 - \exp\left[\dfrac{1-Q_{al}}{Q}\right]} = \ln(Q) - \gamma = I^* - \gamma \quad \text{(S47)}$$

As illustrated by Supplementary Figure 3 and Equation S47, in intermediate regime I_m runs parallel to I^* and the difference between them is γ.

Finally, in activationless regime $Q >> Q_{al}$, both $E_1(1/Q)$ and $E_1(Q_{al}/Q)$ can be approximated according to Equation S45, furthermore $\exp[1/Q] \to 1$ and $\exp[(1-Q_{al})/Q] \approx 1 + (1-Q_{al})/Q$

$$I_m \approx \frac{\ln(Q) - \gamma + \dfrac{1}{Q} - \left(\ln(Q) - \gamma - \ln(Q_{al}) + \dfrac{Q_{al}}{Q}\right) - \ln(Q_{al})\left[1 + \dfrac{1-Q_{al}}{Q}\right]}{-\left[\dfrac{1-Q_{al}}{Q}\right]} \approx \ln(Q_{al}) - 1 \quad \text{(S48)}$$

As illustrated by Supplementary Figure 3 and Equation S48, in activationless regime I_m is saturated at the value 1 unit below I^*.

1.6 Temperature dependence of model parameters

Taking into account the fact that we compare our results with the experimental results measured at room as well as at cryogenic temperatures, it is very instructive to consider what the rate theory model itself tells about the temperature dependence of model parameters. As noted above, we can assume that the attempt frequency ω_a, the activation energy in the absence of the load $E_a(l_0)$, and the factor describing the decrease of E_a with the stretching distance, α_l, are determined solely by the

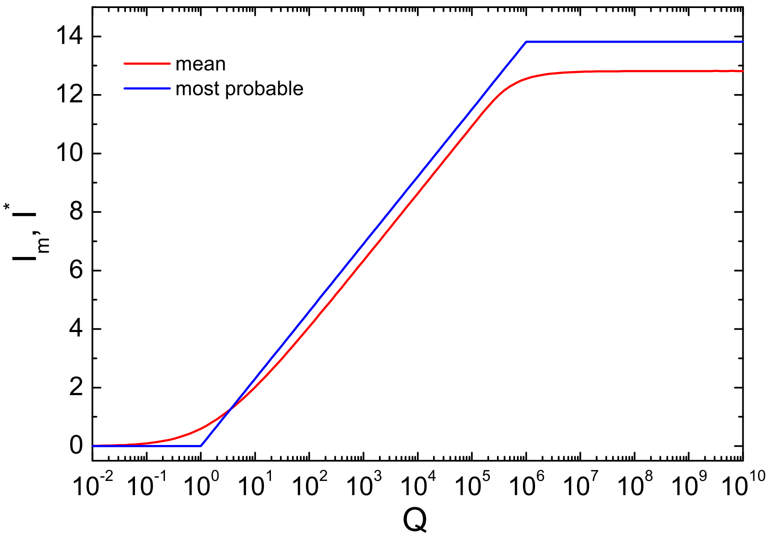

Supplementary Figure 3: Dimensionless functions I^* and I_m calculated as a function of Q for $Q_{al} = 10^6$.

energy landscape of the reaction and therefore temperature-independent. Additionally, the effective spring constant k_s is a system property that is controlled by factors other than temperature and can be considered to be temperature-independent as well. Thus, two model parameters l_{al} and F_{al} which are expressed as combinations of temperature-independent model parameters (Equations S23 and S24), are also temperature-independent. It means that for the same system the same breaking force should be measured in the activationless limit at any temperature, and it may serve as a common reference for data measured at different temperatures.

The most probable stretching distance and breaking force achieve largest possible values l_{al} and F_{al} at the stretching rate v_{al}. The latter is proportional to temperature (Equation S26), which is a weak temperature dependence in the discussed context.

The strongest temperature dependence is exhibited by critical stretching rate v_c, which is proportional to temperature and the rate constant of bond breaking R_0 (Equation S14). The latter is assumed to show Arrhenius-type dependence and may vary by many orders of magnitude with the change of temperature. This result is qualitatively straightforward, as v_c marks transition between the regime of spontaneous, thermally-activated bond breaking and the force-assisted regime, where the bond breaking is accelerated by the mechanical impact.

1.7 Conclusion

The formulated rate theory model gives a very general description of dynamic bond breaking process during stretching in three characteristic regimes: spontaneous, force-assisted and activationless. We assumed a simple linear decrease of the activation energy with the stretching length, avoiding explicit treatment of the reaction's energy landscape[3,4], as well as ignoring the possibility of rebinding[5]. To the best of our knowledge, it is the first theoretical framework that integrates and explicitly treats the activationless bond breaking regime. In the presented framework, a system is completely characterized by three parameters: activation energy in the absence of applied load $E_a(l_0)$, the coefficient showing the decrease of activation energy with the stretching length α_l and attempt frequency ω_a (or the rate constant of the spontaneous breaking at one temperature R_0). The breaking force can be quantified if the spring constant of the system is known. Furthermore, the model explicitly treats temperature, allowing to predict temperature effect on dynamic bond breaking process or compare results obtained at different temperatures. Besides typically treated most probable stretching time, stretching length and breaking force[1], the equations for mean values were derived as well. The latter shows a similar behavior in three characteristic regimes, but show smooth transitions between regimes that better resemble experimental curves.

Supplementary Note 2: Experiment

2.1 Experimental and analysis details

Current-sensing force spectroscopy experiments were carried out using a CSAFM setup based on the PicoPlus 5500 SPM system (Agilent Technologies)[6]. Unless stated otherwise, the experimental and analysis details were the same as in Ref. 6.

As AFM probes we used three types of commercial gold-coated Si cantilevers (Nanosensors) with spring constants $k \approx 5$ N·m^{-1} (PPP-NCSTAu), $k \approx 10$ N·m^{-1} (PPP-NCSTAu, special selection) and $k \approx 40$ N·m^{-1} (PPP-NCLAu). Data sampling rate varied from 10 to 100 kHz. All presented experiments were carried out in decane (99%, Sigma-Aldrich). The parameters of all experiments are summarized in Table 1.

Processing of experimental data was implemented in a CYGWIN environment running under Windows OS using a combination of shell scripts, standard CYGWIN programs and self-written C programs (the source codes of the programs are available upon request to Ilya V. Pobelov).

The continuous experimental files containing time, applied piezo voltage, cantilever deflection and the current flowing through the junction were first separated into individual time-deflection-conductance traces. The piezo voltage signal was adjacent-averaged with a radius of 5 ms. Then its time derivative was calculated by taking the slope over a part of the trace including the data points within 2 ms before and after a given point. The time segments corresponding to the absolute value of the probe movement rate $|v|$ being below 1 nm·s^{-1} were attributed to a stationary probe and discarded. The other time segments corresponding to the moving probe were sorted into approaching and withdrawing traces, based on the sign of the first derivative of the piezo voltage. The absolute value of the junction conductance G was calculated from the measured current and the applied bias voltage 0.13 V and normalized with respect to the quantum conductance $G_0 = 77.5\ \mu$S. The individual time-deflection-conductance traces were produced at this stage. Only withdrawing traces displaying the evolution of the conductance from $G > 10\ G_0$ to $G < 10^{-6}\ G_0$ were processed further.

Each "full" trace obtained at the previous step was aligned as following. The baseline of the cantilever deflection $V_{d,0}$ was determined as an average value of V_d measured in time interval from 10 to 20 ms after reaching the conductance $10^{-6}\ G_0$. Traces, which did not have sufficient data for the calculation of the baseline, were rejected at this stage. The variation of the deflection $\Delta V_d = V_d - V_{d,0}$ was converted to the force F using the experimentally determined spring constant k and the deflection sensitivity. The zero of the time scale was assigned to the first point, where the junction conductance G dropped below 0.1 G_0. Only data from no more than -0.2 s in the new time scale and until the end of the force baseline range were used to produce an aligned trace.

2.2 Individual traces

Supplementary Figure 4 shows typical traces illustrating the evolution of pulling force and conductance upon stretching of gold nanocontacts with $v = 10$ nm·s^{-1}. The spring constant of the cantilever was $k = 5.969$ N·m^{-1}. The representation of the forces follows the convention of a negative (positive)

Supplementary Table 1: Parameters of the dynamic force spectroscopy experiments.

cantilever spring constant k (N·m^{-1})	stretching rate v (nm·s^{-1})	force loading rate r_f (nN·s^{-1})	sampling rate kHz
5.32	5	26.6	10
5.97	10	59.7	20
5.25	50	262.5	20
5.44	100	544	100
4.64	200	928	100
10.11	100	1011	100
9.69	200	1938	100
11.51	500	5755	100
11.38	1000	11380	100
11.73	2000	23460	100
12.70	3000	38100	100
38.5	5	192.5	20
45.5	10	455	10
39.4	20	788	20
38.4	50	1920	20
33.6	100	3360	20
38.4	200	7680	20

sign for attractive (repulsive) interactions, and zero in the absence of interactions. The traces were obtained as described in Supplementary Note 2.1 and presented without any smoothing, resampling or other type of data processing. The noise on force curves is similar to or lower than previously observed in experiments of this type at room temperature[7–11] (see also discussion in Ref. 6). To facilitate representation of conductance G in a wide range (7–8 orders of magnitude) of measured values, we converted it into the G' scale defined as[6]:

$$\begin{aligned} G' &= G/G_0 \text{ if } G \geq G_0 \\ &= 1 + \ln\left(G/G_0\right) \text{ if } G < G_0 \end{aligned} \tag{S49}$$

G' is linear for $G \geq G_0$, the typical conductance range for atomic contacts, and logarithmic for $G < G_0$, a conductance range that is characteristic for molecular junctions.

Atomically-thin gold contacts created by stretching junctions are known to demonstrate well-defined features of quantized conductance $G \approx N \cdot G_0$ (Ref. 12). A typical sequence of the stepwise formation of gold atomic contacts and their transformations upon stretching is shown by trace 1. The individual segments corresponding to (meta)stable contacts are characterized by a slowly varying loading force (elastic deformation) and an almost constant conductance of integer multiples of the G_0. They are separated by contact yielding events, as represented by a sharp decrease of the absolute force value and a simultaneous decrease in contact conductance. The formation of a single-atom gold contact is easily identified by its conductance G amounting to $\approx 1\,G_0$. After the contact is completely broken, the measured conductance drops to the lower detection limit (background conductance) of the experimental setup ($G' < -11$ or $G < 10^{-5}\,G_0$), which indicates the absence of an electric contact.

We measured several thousands of individual force and conductance traces with the same cantilever using an identical stretching rate, and statistically evaluated the obtained data sets to obtain representative characteristics. In addition to clearly resolved traces of formation and breaking of gold

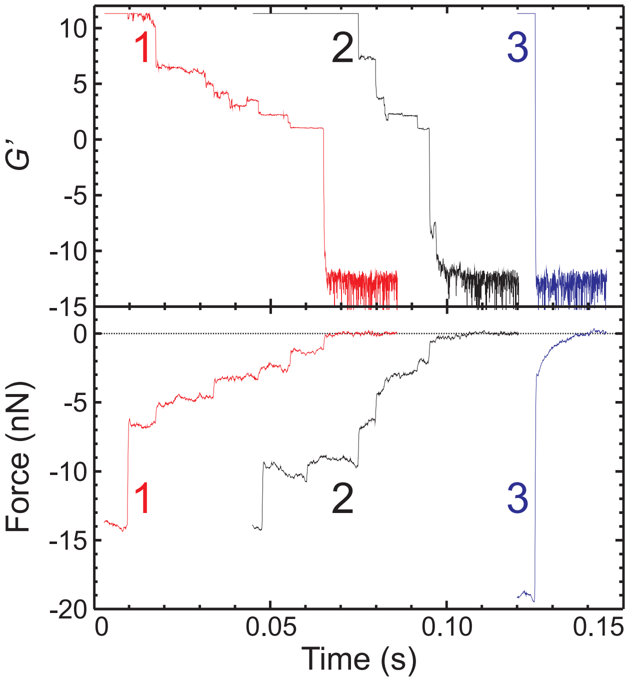

Supplementary Figure 4: Examples of individual conductance and force vs. time withdrawing traces recorded with a gold-coated cantilever and a gold sample in decane ($k = 5.969$ N·m^{-1}, $v = 10$ nm·s^{-1}). Trace 1 demonstrates formation of single-atom gold contact and its clean breaking; trace 2 shows an additional feature between the conductance of gold contacts and the background level; trace 3 illustrates an abrupt contact rupture.

contacts (trace 1 in Supplementary Figure 4), we also observed two other types of traces. In one case (trace 2 in Supplementary Figure 4) the drop of the conductance to the background conductance is not as smooth as for the traces of the first type, and additional features are found between the conductance of the gold nanocontacts and the background conductance. Their occurrence is attributed either to a mechanical instability of the junction or to the formation and breaking of an atomic junctions containing impurities. The third type of traces (trace 3 in Supplementary Figure 4) is characterized by an abrupt rupture of gold nanocontacts with $G > 11\ G_0$ accompanied by large change in force (≥ 15 nN). Traces of the third type do not contain any information of interest. Therefore prior to the further analysis we removed from the experimental data sets traces showing the decrease of the conductance from $G > 10.5\ G_0$ to $G < 0.1\ G_0$ in less than 0.1 ms.

2.3 Histograms

Subsequently, we constructed all-data point histograms from traces belonging to one data set to evaluate the characteristic features of the stretched gold nanocontacts. Supplementary Figure 5 illustrates results for a typical data set recorded with $k = 5.969$ N·m^{-1} and $v = 10$ nm·s^{-1}. The 1D conductance histogram in Supplementary Figure 5a is plotted in the G' representation. The formation of single-atom gold contacts is represented by the main peak at $G' = 1$. Additional peaks at higher $G' = 2,3,4,\ldots$, indicate the formation of other gold nanocontacts with quantized conductance. The wide peak around $G' = -12.15$ ($G \approx 2 \times 10^{-6}\ G_0$) marks the background level of conductance in our setup. The sharp decrease of the conductance after breaking of the atomic contacts results in a negligible amount of data points in the range $-5 < G' < 0$. The counts between -5 and the background conductance peak are related to the tunnelling and possible contribution of contaminations.

1D histograms of this type, as commonly used to determine preferable conductance of nanojunctions, indicate the formation of gold nanocontacts with a characteristic quantized conductance, but neglect time and force information. Therefore, we constructed 2D histograms of conductance and force by calculating the occurrence of (G', t) and (F, t) pairs from all aligned traces in the $G' - t$ and $F - t$ fields. The 2D conductance histogram in Supplementary Figure 5b demonstrates several distinct lines of high data density at $G' > 0$, such as at $G' = 1,2,3,\ldots$. They represent the formation of gold contacts with quantized conductances before the contact breaking ($t < 0$). A single cloud-like feature developing after the contact breaking ($t > 0$) at $G' < -11$ corresponds to the background level of the measured conductance (c.f. Supplementary Figure 4). It indicate that for the majority of traces the conductance quickly drops to the detection limit after the contact break. We also observed a certain amount of data points in the range $-11 < G' < -5$, which originate from those traces with contributions of contaminations (type 2 in Supplementary Figure 4). A comparison of the 2D force histogram (Supplementary Figure 5c) with 2D conductance histogram (Supplementary Figure 5b) allows attributing the cloud of force values between -4 and -1 nN to the stretching of small gold contacts. Individual contributions could not be separated at this stage. The mechanical relaxation of the cantilever after breaking the contact appears to produce a broad tail of force values at $t > 0$. Similar histograms were obtained for other data sets (Table 1).

2.4 Separation of "clean" traces

In the next step we removed traces with possible contributions of contaminations, i.e. the traces displaying long plateaus with conductance below 1 G_0 (c.f. trace 2 in Supplementary Figure 4). We found for each trace the time t_n, during which the conductance stays above the baseline level after the contact break, by determining the position of the last point with a conductance $G' > -10$ ($G > 1.67 \times 10^{-5}\ G_0$) in the aligned time scale. The value $G' \approx -10$ corresponds well to the offset of the baseline conductance peak for all measured data sets.

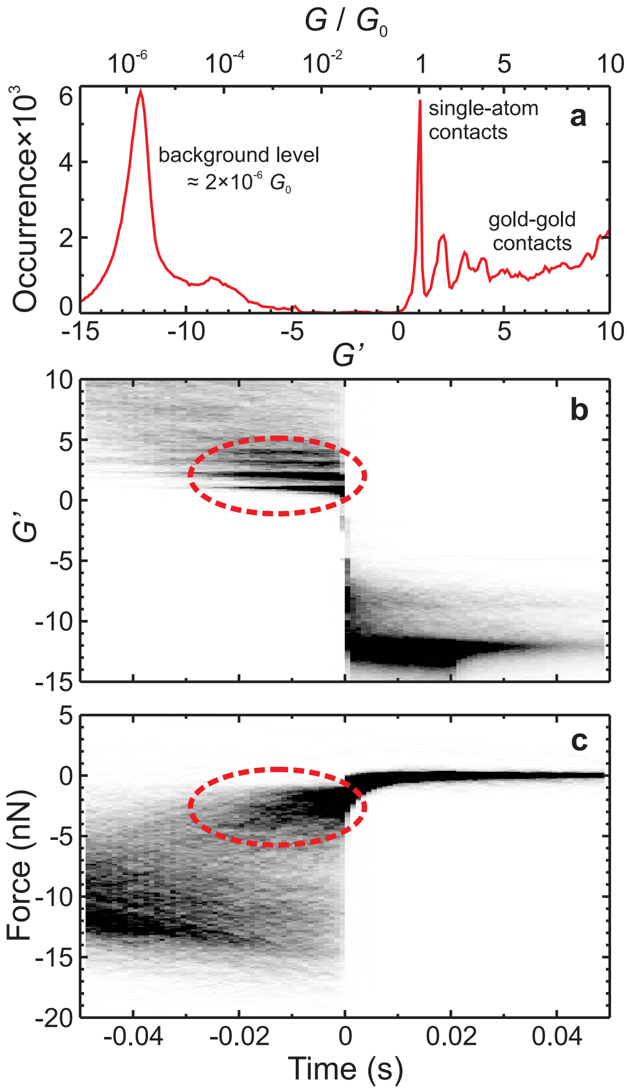

Supplementary Figure 5: Histograms constructed from 2042 traces, which have been measured under the same experimental conditions as the traces shown in Supplementary Figure 4. The data plotted represent the ratio of the number of data points within a given bin normalized with respect to the total number of data points in all traces. (a) 1D conductance histogram in the G'-representation, bin size 0.1. The top scale shows the corresponding values of G/G_0. (b,c) 2D $G' - t$ (b) and $F - t$ (c) histograms. The bin sizes are 0.1×1 ms and 0.1 nN$\times$1 ms, respectively. Color scale corresponds to the variation of the occurrence from 0 (white) to $\geq 5 \times 10^{-5}$ (black). The red circles mark features in the 2D histograms indicating the formation of gold atomic contacts.

The histograms of t_n for the sets of traces measured with cantilevers having spring constants of $k \approx 5$ N·m^{-1} and $k \approx 10$ N·m^{-1} are shown in Supplementary Figure 6. They demonstrate that for the majority of data sets the peak of t_n is found within 0.5 ms after the contact break, independently on the stretching rate. This is illustrated by Supplementary Figure 7, which shows a 2D conductance-time histograms of all traces measured with cantilevers having spring constants of $k \approx 5$ N·m^{-1} using the stretching rate of 10 and 100 nm·s^{-1}. As one can clearly see, the appearance of histogram just after the contact break ($t = 0$) is invariant if the time is used as an independent variable. We interpret it as an indication that in the absence of contaminations the process responsible for the finite time of conductance decay to the background level is an intrinsic, stretching rate independent relaxation of the cantilever (see discussion below). We further discarded the traces with $t_n > 1$ ms.

On the other hand, the data sets measured by cantilevers with $k \approx 40$ N·m^{-1} displayed much

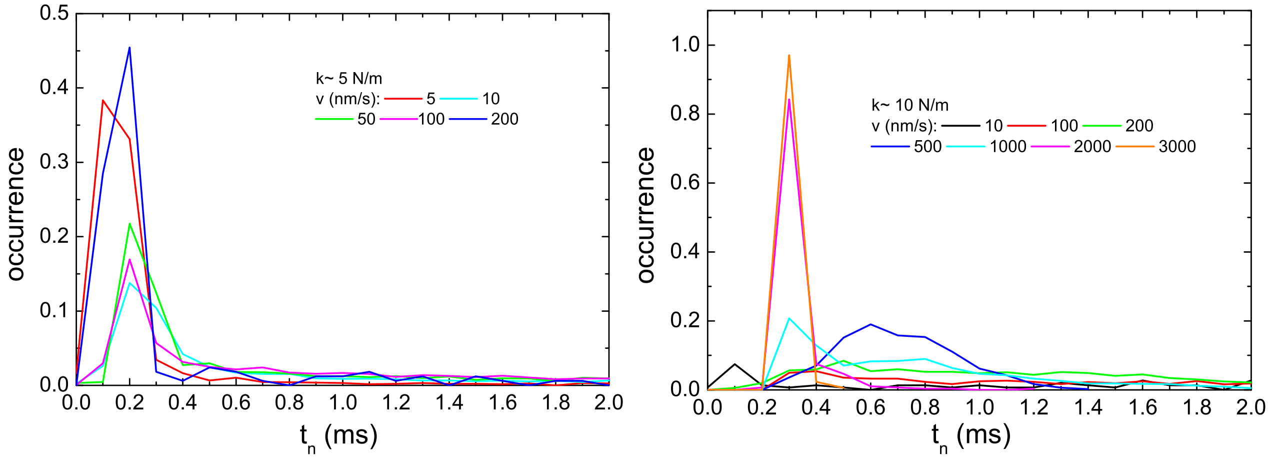

Supplementary Figure 6: Histogram of t_n calculated for all data sets measured with cantilevers having a spring constant $k \approx 5$ N·m^{-1} and $k \approx 10$ N·m^{-1}. The bin width is 0.1 ms.

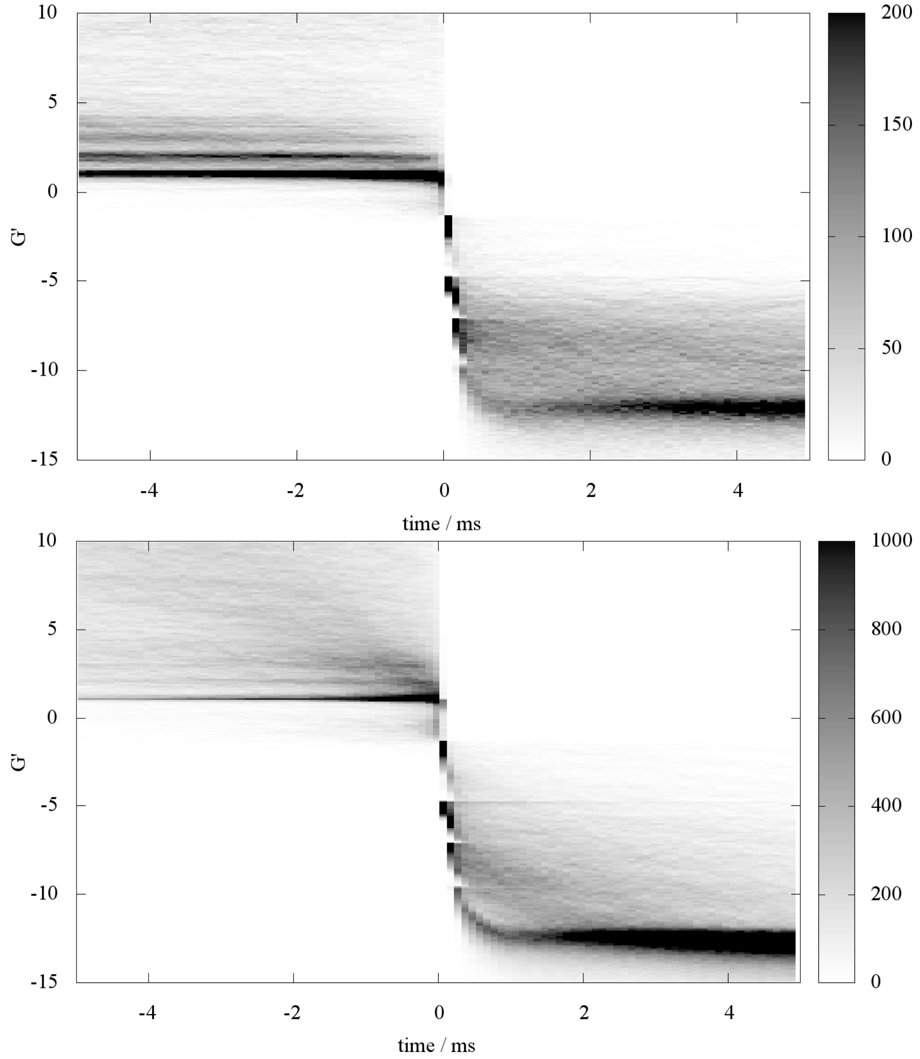

Supplementary Figure 7: 2D conductance-time histogram of all aligned traces measured with cantilevers having $k \approx 5$ N·m^{-1} using the stretching rate of 10 (top) and 100 (bottom) nm·s^{-1}. Bin size is 0.1×0.1 ms, color scale displays variation of the number of counts in each bin.

higher t_n than for the other cantilevers. We attribute this difference to the contribution of tunnelling current measured after the breaking of the gold-gold contact. The distributions of distance to baseline level $d_n = v \cdot t_n$ were invariant in this case, with a peak at 0.1-0.2 nm (Supplementary Figure 8). This is illustrated by Supplementary Figure 9, which shows a 2D conductance-stretching distance histograms of all traces measured with cantilevers having spring constants of $k \approx 40$ N·m^{-1} using the stretching rate of 10 and 100 nm·s^{-1}. After the contact break ($t = 0$), the conductance is rapidly decreasing to $G' \approx -5$ ($G \approx 2.5 \times 10^{-3}$ G_0), and then decays exponentially (reflected as a linear decay in G' scale) until the background level of conductance is reached. This is a typical conductance-distance dependence due to the tunnelling between electrodes as observed in break-junction type experiments in a pure solvent without target molecules. For comparison and a detailed discussion of observed conductance-distance dependence we refer to Ref. 13. It is known that the effective spring constant of electrodes used in break-junction experiments is very high, therefore the latter are better comparable with CSAFM experiments employing hard cantilevers. In case of softer cantilevers, after the contact break there is a significant set-back, i.e. an intrinsic movement of the cantilever away from the sample not related to the hardware-controlled probe displacement. It quickly brings the probe and the sample out of the tunnelling contact and produces conductance-time dependences discussed above. Based on the appearance of the histograms we selected 0.5 nm as a threshold and discarded traces with $d_n > 0.5$ nm.

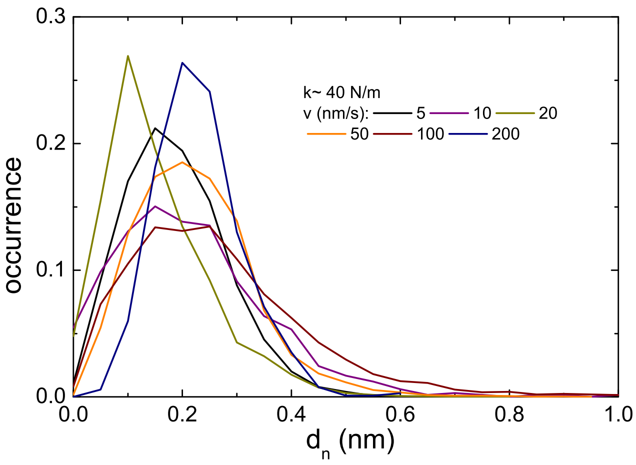

Supplementary Figure 8: Histogram of d_n calculated for all data sets measured with cantilevers having a spring constant $k \approx 40$ N·m^{-1}. The bin width is 0.05 nm.

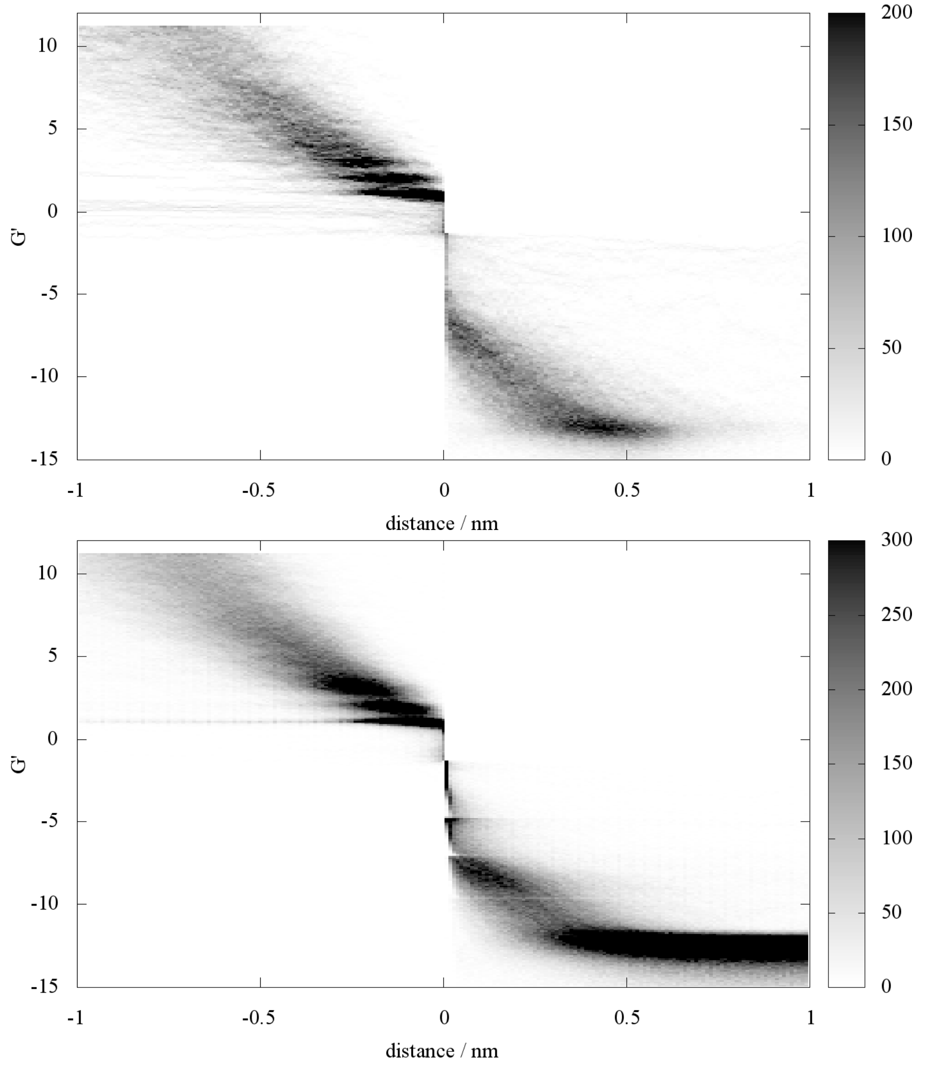

Supplementary Figure 9: 2D conductance-stretching distance histogram of all aligned traces measured with cantilevers having $k \approx 40$ N·m^{-1} using the stretching rate of 10 (top) and 100 (bottom) nm·s^{-1}. Bin size is 0.1×0.01 nm, color scale displays variation of the number of counts in each bin.

2.5 Separation of subsets of traces

Further we selected subsets of traces corresponding to the breaking of gold nanocontacts with defined conductance. A conductance plateau corresponding to the formation of a gold atomic contact with conductance $G \approx N \cdot G_0$, $N = 1, 2, \ldots, 10$, was defined as a part of the trace between the first point with a conductance $G_{first} < (N + 0.5)\ G_0$ and the last point with a conductance $G_{last} > (N - 0.5)\ G_0$. After the first and the last point of the conductance plateau were found for a given trace, the difference of the force values corresponding to these points was checked. If $F_{last} - F_{first}$ was higher than 1 nN, the first point of the plateau was shifted further until this condition was satisfied. An additional check of the force values was necessary to select segments of traces without an initial sharp increase of force due to the yielding of the previous configuration.

The plateau length data can be treated either using the time value $t_N = (t_{N,last} - t_{N,first})$ or using the distance value $d_N = v \cdot t_N$. Supplementary Figure 10 demonstrate the histograms of the plateau length for single-atom gold-gold contacts, as obtained under all conditions studied. For traces measured with a stretching rate ≤ 1000 nm·s^{-1} the histograms correspond to each other better if the distance value is used. For faster experiments, the distributions of time rather than of distance were more similar. Based on the appearance of histogram and test runs, we found that a selection of traces with $d_N > 0.05$ nm (or $t_N > 0.05$ ms for fast experiments) is sufficient to obtain force-distance and conductance-distance histograms with well-defined features corresponding to the breaking of a specific type of contact.

As a last selection criterion, we considered the position of the last point of the conductance plateau in the aligned scale. The purpose of this check is to select the traces with plateaus followed by the

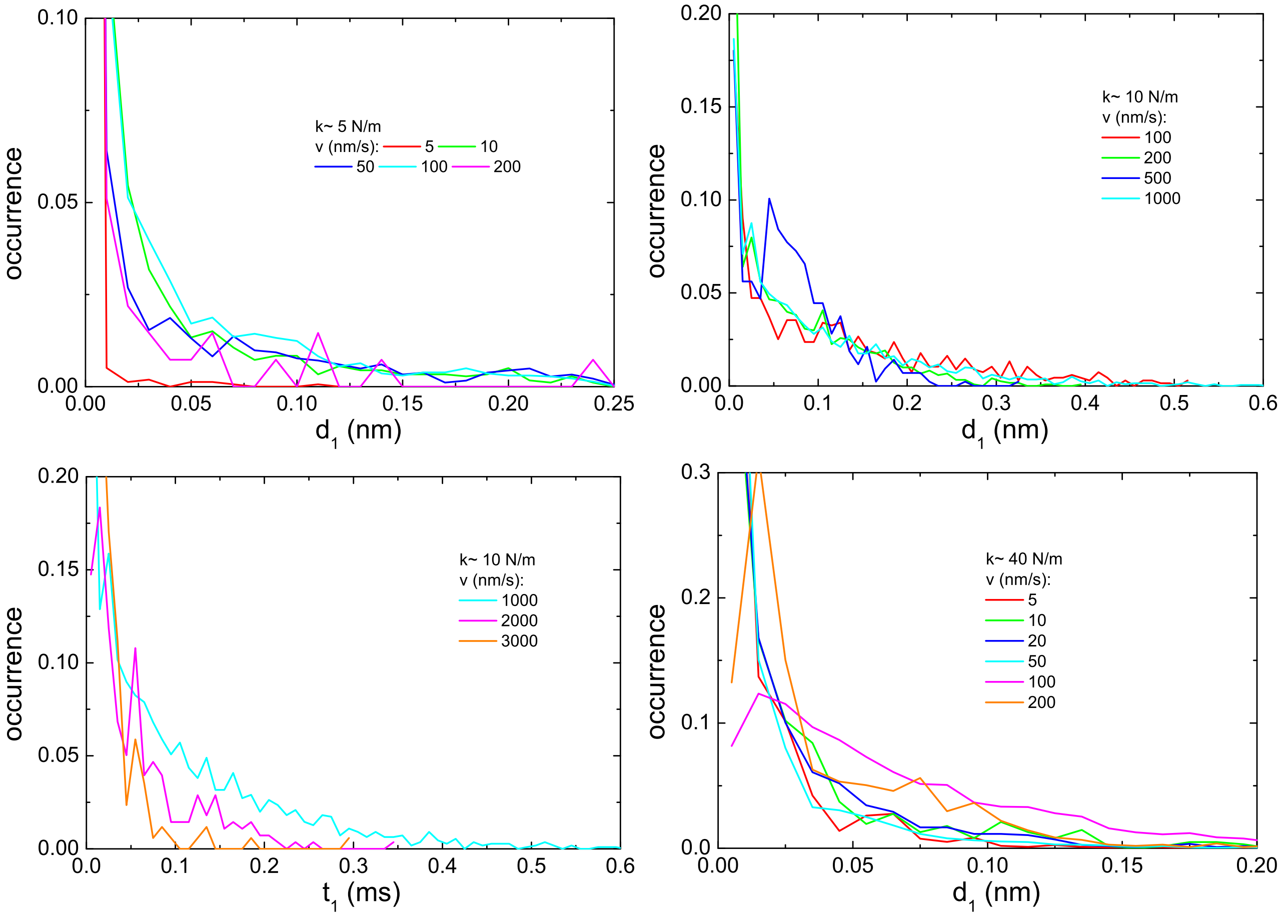

Supplementary Figure 10: Histogram of d_1 and t_1 calculated for all measured data sets. The bin width is 0.01 nm.

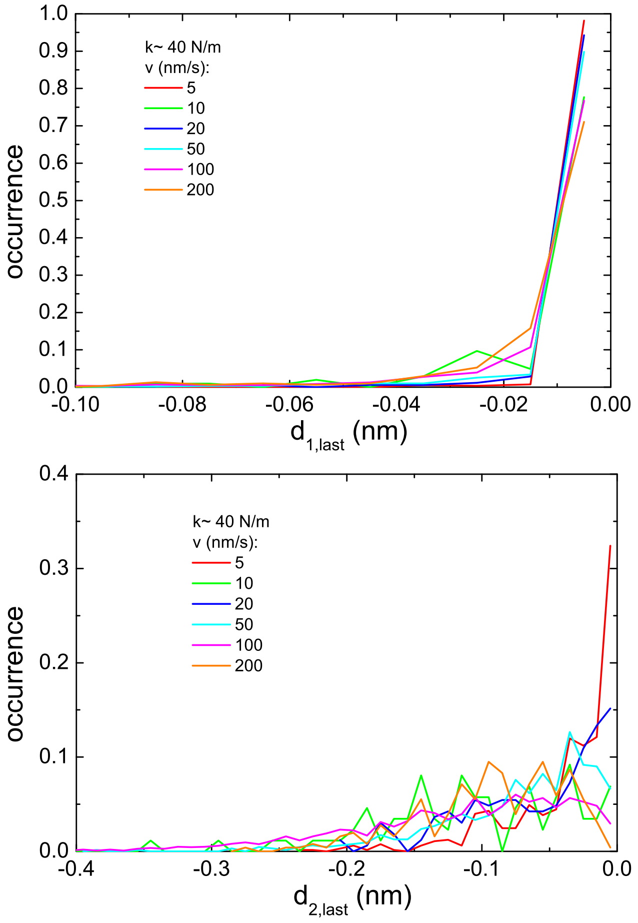

Supplementary Figure 11: Histogram of $d_{1,last}$ and $d_{2,last}$ for selected plateaus with a conductance $G \approx G_0$ and $G \approx 2\ G_0$ as extracted from all data sets measured with cantilevers having a spring constant $k \approx 40$ N·m^{-1}. The bin width is 0.01 nm.

contact break, and not by a transition to another contact. Supplementary Figure 11 shows distribution of the position of the last point of the conductance plateau $d_{last} = v \cdot t_{last}$ with conductances $G \approx G_0$ and $G \approx 2\ G_0$ selected as described above the data sets measured with hard cantilevers. At least 70% of the selected single-atom plateaus finish between -0.01 nm and 0 of the aligned scale. In contrast, the majority of plateaus with a conductance $G \approx 2\ G_0$ finishes much farther from the contact break. This is due to the fact that gold contacts created with hard cantilevers almost always form single-atom gold contacts before breaking. Therefore, we selected only traces with conductance plateau finishing not more than 0.01 nm (0.01 ms for fast experiments) before the contact break.

2.6 Calculation of breaking force

Subsets of the traces, identified according to the above criteria, were realigned by taking the last point of the conductance plateau as new zero of the distance and of the force scales. Then, the 2D conductance and force histograms for a given subset of traces were constructed.

Supplementary Figure 12 displays the 2D histograms as obtained for a subset of traces extracted from data displayed in Supplementary Figure 5, which represent the breaking of a single gold-gold bond. Both histograms display only one continuous band, which can be represented as a statistically averaged mean trace. The latter was obtained by fitting every vertical column of the 2D histogram (i.e., the occurrence vs. G' or F at fixed t) with a Gaussian. Position and standard deviation of the peak represent the most probable value of the corresponding variable, G'_m and F_m, and its error. The

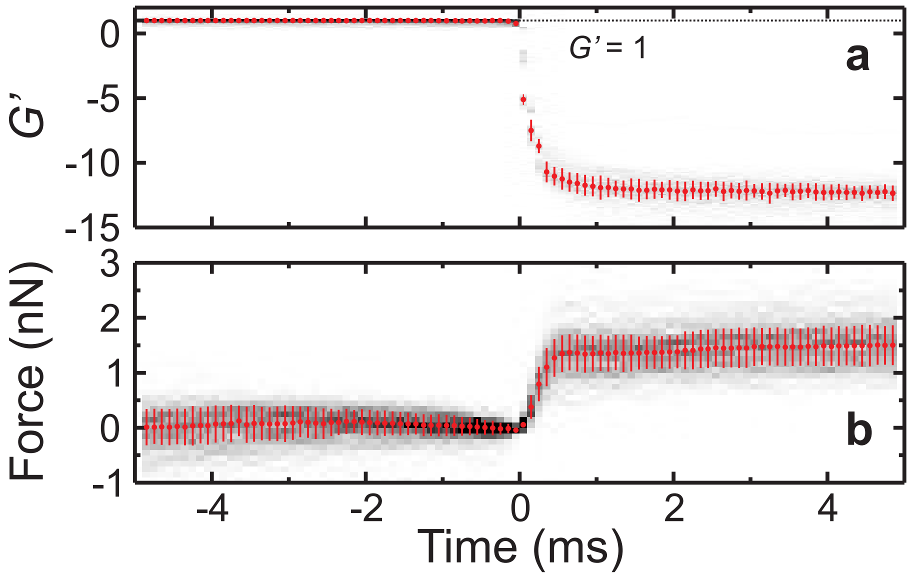

Supplementary Figure 12: Histograms of 164 traces, extracted from the data set presented in Supplementary Figure 5, which displays the evolution of (a) conductance and (b) force after breaking of single-atom gold-gold contacts. The bin sizes are 0.1×0.1 ms and 0.1 nN$\times$0.1 ms, respectively. The color scale corresponds to the variation of the occurrence from 0 (white) to $\geq 10^{-4}$ (black). The circles and the vertical lines indicate the mean value and the standard deviation of the corresponding data at fixed t. The former may be considered as the most probable values of conductance or force at a given time.

result is plotted as dots and vertical lines in Supplementary Figure 12. The slope of the mean force versus time trace in Supplementary Figure 12b prior to breaking the contact (apparent force loading rate) equals to 60.7 nN$\cdot$s^{-1}, and corresponds very well to the formal force loading rate $r_f = k \cdot v = 59.7$ nN$\cdot$s^{-1}.

The evolution of mean force traces after the contact break reveals a steady force increase followed by a rather constant force, as exemplified by Supplementary Figure 12b. The force attained after the initial steady increase and its standard deviation were attributed to the mean value of the bond breaking force under given experimental conditions and its error. The resulting breaking force for single-atom gold contacts is summarized in Figure 2b. Several experiments allowed extracting subsets of traces corresponding to the breaking of gold atomic contacts with $G \approx N G_0$ for $N > 1$ (Figure 2a). Taking into account the magnitude of the error bar, the presented values of the breaking force are robust with respect to the exact position of the point used to determine it as well as to the exact position of "the last point of the conductance plateau" used to realign the traces.

Evaluation of the mean force traces for all experiments demonstrated that the time interval of steadily increasing force is independent of v or r_f as well as of the amount of gold atoms in the cross-section, and varies between 0.4 to 0.6 ms across the mean traces from different experiments. Tao et al. suggested in a study employing an ac-modulation technique that the breaking of gold-gold monatomic contacts proceeds at a picoseconds time scale (< 0.1 ns),[14] i.e. many orders of magnitudes faster than the "rise time" of $\approx$0.5 ms observed in our experiments. A possible explanation for the finite rise time of the force immediately after the contact break is an intrinsic, stretching rate independent relaxation of the cantilever in the liquid environment. In this sense, the finite rise time gives a measure The force acting on the cantilever often shows a slow non-systematic increase after 0.5 ms, which may be related to long-range force interactions.

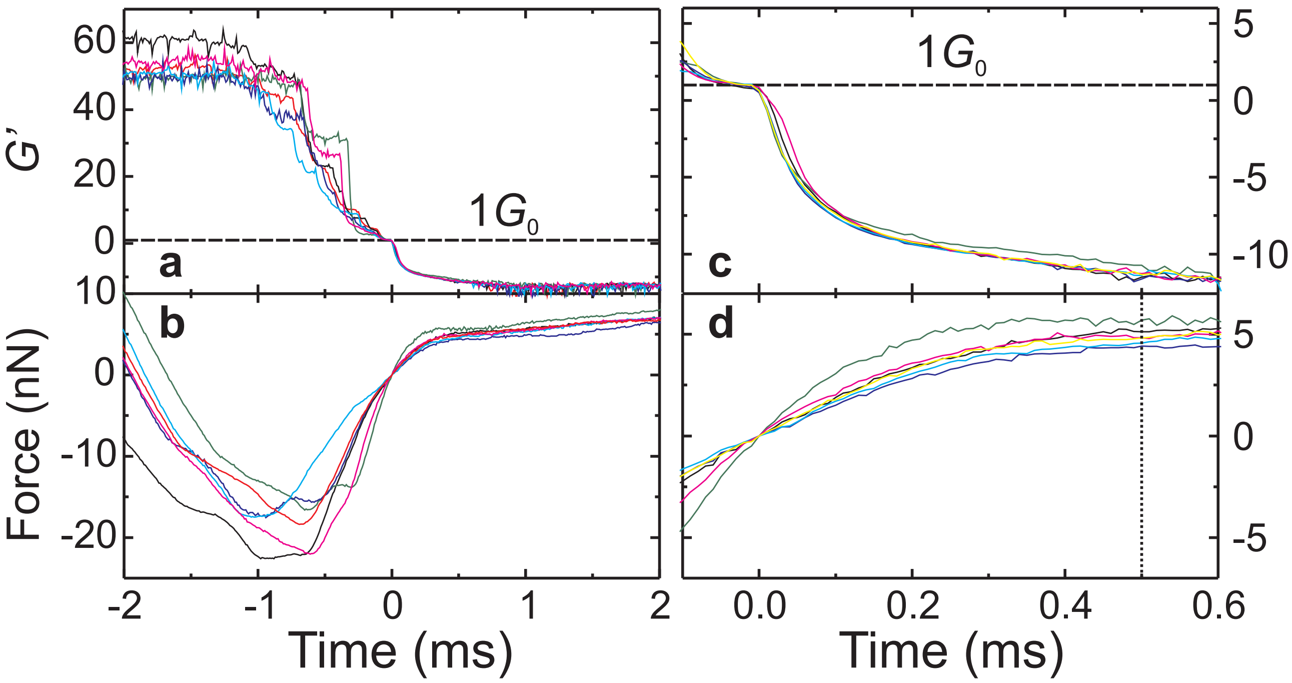

Supplementary Figure 13: Examples of individual conductance and force vs. time withdrawing traces showing formation of plateaus with conductance $1G_0$ in experiments with stretching rate $v = 3000$ nm·s^{-1} ($k = 12.7$ N·m^{-1}). The corresponding conductance and force traces are shown by the same color. The horizontal dashed lines in panels (a) and (c) mark conductance equal to 1 G_0; the vertical dotted line in panel (d) marks the position used to determine mean breaking force. Panels (c) and (d) are zooms of data shown in panels (a) and (b), respectively.

2.7 Measurements at high stretching rates

Finally, we would like to discuss results obtained at high stretching rates separately. Supplementary Figure 13 displays few traces measured with the highest stretching rate employed, $v = 3000$ nm·s^{-1}, that shows plateaus with conductance $G \approx 1G_0$ and length $t_1 > 0.05$ ms (Supplementary Note 2.5). The traces were realigned so that the last point of conductance plateau corresponds to zero of the distance and of the force scales (Supplementary Note 2.6). All traces show adhesive behaviour at $t < -1$ ms, with a rather constant conductance (Supplementary Figure 13a) and decreasing force (Supplementary Figure 13b) upon pulling. This behaviour can be explained by the probe "sticking" to the substrate after a contact formation. When the pulling force reaches -15 to -20 nN in this scale, the gold-gold contact neck starts to yield and shrink, as indicated by the decrease of conductance and increase of force. Contacts relaxation and shrinking proceeds until a contact with conductance $G \approx 1G_0$ forms, as seen on conductance traces (Supplementary Figure 13c). After contact breaking ($t = 0$), the force signal shows a relaxation to ≈5 nN within 0.5 ms, as marked by the vertical dotted line in Supplementary Figure 13d. After averaging all selected traces according to the procedure outlined above (Supplementary Note 2.6), we obtained the formal value of breaking force attained at 0.5 ms after the contact break equal to 5 ± 0.9 nN.

However, we would like to point out that, unlike traces measured at low stretching rates (Supplementary Figure 4), the curves in Supplementary Figure 13 do not show linear segments on force traces corresponding to $1G_0$ plateaus on conductance traces. This might indicate two phenomena. First, the stability time of $1G_0$ contacts estimated from conductance traces, 0.05 to 0.1 ms, could be insufficient to create "relaxed" contacts. The non-relaxed contacts created during the fast stretching may exhibit different properties than contacts created at lower stretching rates and showing corresponding linear segments on force traces. Second, the absence of the linear segments on force traces corresponding

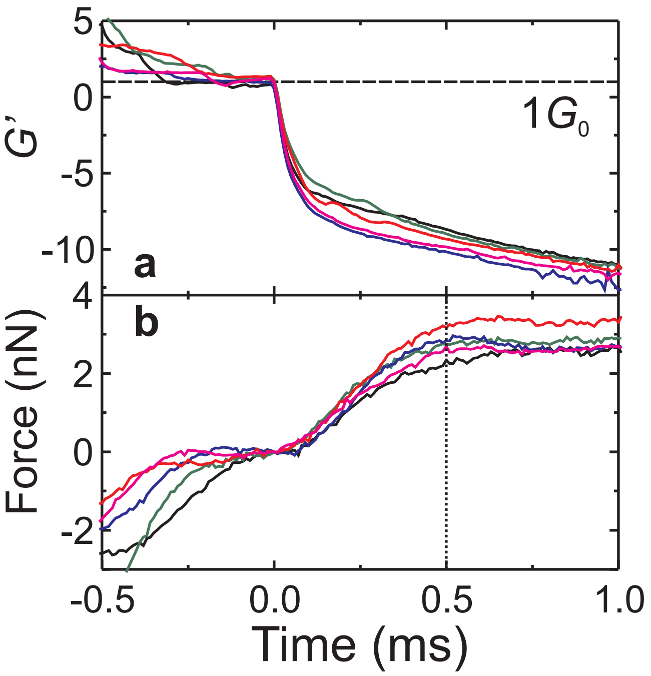

Supplementary Figure 14: Examples of individual conductance (a) and force (b) vs. time withdrawing traces showing formation and breaking of single-atom gold contacts in experiments with stretching rate $v = 1000$ nm·s^{-1} ($k = 11.4$ N·m^{-1}). The corresponding conductance and force traces are shown by the same color. The horizontal dashed line in the panel (a) marks conductance equal to 1 G_0; the vertical dotted line in panel (b) marks the position used to determine mean breaking force.

to $1G_0$ plateaus on conductance traces might indicate that the observed force evolution is not related to the breaking of single-atom contact, but it is rather a part of a force relaxation transients produced after the breaking of larger contacts. Therefore, to avoid possible complications, for stretching rates > 200 nm·s^{-1} we explicitly considered only force-conductance traces that showed on force traces linear segments correlated with $1G_0$ conductance plateaus. Such traces were selected by visual inspection of individual traces selected during the previous analysis steps (Supplementary Notes 2.4 and 2.5). For stretching rates ≤ 200 nm·s^{-1} this requirement was found to be already fulfilled.

For experiments carried out with the stretching rate of 3000 and 2000 nm·s^{-1}, we did not find force-conductance traces that unambiguously fulfill later requirement. On the other hand, the traces measured with the stretching rate of 1000 nm·s^{-1} showed linear force segments correlated with $1G_0$ conductance plateaus. 40% of previous chosen traces have been selected at this stage for this data set. Few examples of selected traces are shown in Supplementary Figure 14. After the contact break, the curves in Supplementary Figure 14b shows increase of the force to values above 2 nN, which is clearly above the value of 1.5 nN commonly quoted as the breaking force of the single-atom gold contact. The breaking force obtained after averaging of all selected traces was 3.3 ± 0.7 nN, as shown by the right-most point in Figure 2b. For the data set measured with the stretching rate of 500 nm·s^{-1}, the selection ratio at the last step was 36% and the obtained breaking force is shown as the second right-most point in Figure 2b.

A single dependence of breaking force vs. force loading rate r_f is obtained when compiling experimentally obtained breaking forces of single-atom gold contacts for a wide range of stretching rates (5 to 1000 nm·s^{-1}) and three types of cantilevers (Figure 2b). For gold contacts with $N = 2$, the breaking force vs. r_f dependence seems to show an increase of the breaking force at the upper limit (Supplementary Figure 15), but the point of transition to the force-assisted regime cannot be well identified. For higher N, the trends are even less clear.

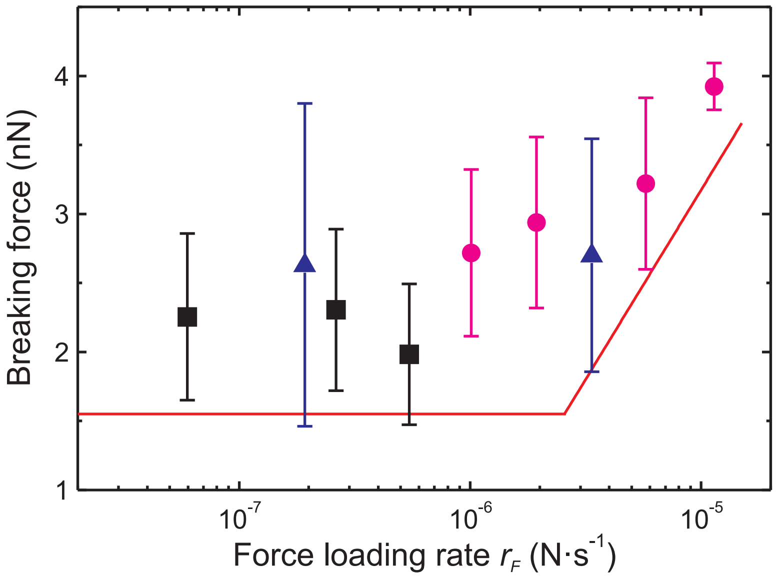

Supplementary Figure 15: Mean value (symbols) and the standard deviation (error bar) of the breaking force of the gold contacts with conductance $G \approx 2G_0$ as obtained in experiment employing cantilevers with spring constants $k \approx 5$ N·m^{-1} (■), $k \approx 10$ N·m^{-1} (●) and $k \approx 40$ N·m^{-1} (▲). The red lines are trend lines for the breaking force of single-atom gold contact (c.f. Figure 2b).

From the technical point of view, the fast stretching may lead to signal sampling artefacts. Although unusual for break-junction type conductance measurement experiments, the probe movement rates in the order of few μm·s^{-1} are routinely used in force spectroscopy experiments performed in an AFM setup. The sampling rate of 100 kHz (Table 1) employed in experiments at high stretching rates converts to the sampling interval of 0.01 ms, allowing to resolve details of conductance and force evolution. The rise time of force signal, from the contact break to a stable value, is ≈0.5 ms for all stretching rates, which is a strong indication that the result obtained at high stretching rates are not limited by the bandwidth of the experimental setup. A careful inspection of yielding events on many conductance and force traces measured with the sampling rate of 100 kHz showed that the difference between the last point before the sharp conductance decrease and the inflection point on corresponding force curve is not systematic, can be both negative and positive but more often positive, and never exceeds 0.15 ms. The observed difference can be attributed on one hand to a subjectivity in determining the position of respective points in real, imperfect experimental curves; on the other hand we do not exclude an instrumental factor. Indeed, in the experimental setup the lab-build current amplifier had a short connection to the probe, fast analog to digital converter and a fast digital connection to the data acquisition unit. The force, on the other hand, was measured as an analog signal by a quadrant detector, passed by cables to the break box and then to the data acquisition unit, where it was digitized. The SPM controller must have an analog or digital circuitry that calculates the deflection and other signals from the raw photodiode signals. This circuitry may produce a certain time delay, and if it does not work continuously, the time delay might be also quite random. Such delay might lead to the observed small lag between signals, which is nevertheless significantly smaller than the rise time of the force signal and does not affect our conclusions.

Supplementary Note 3: Theoretical calculations and simulations

3.1 Simulation of individual pulling curves

Using ASE[15] a system of 6 gold atoms on a straight line was created in a cell with 20 Å of vacuum around the chain and with periodic boundary conditions. EMT[16,17] was used as the energy calculator. This system was relaxed using BFGS until the maximal force on any atom was 0.01 eV/Å. The terminal atoms were then constrained in space and the system was thermalised to the desired simulation temperature using the Maxwell-Boltzmann distribution.

To simulate the pulling we used Langevin dynamics as implemented in ASE with a timestep of $\Delta t = 0.5$ fs and a friction constant of 1×10^{-3} inverse atomic units of time.

We were able to simulate a large range of stretching rates, $v = 1 \times 10^{-9}$ m/s to 1×10^{-1} m/s. For a given v the pulling scheme is as follows:

1. The system was allowed to evolve with Langevin dynamics for some number of timesteps, n.
2. The constraints on the terminal atoms were lifted and the rightmost atom was moved a small distance, $d = 1 \times 10^{-14}$ m (to the right).
3. The terminal atoms are constrained again.
4. At regular intervals we test if the chain is broken.
5. If it is broken we run the simulation for 10 ps to allow the breaking to complete.
6. If it is not broken, we go to step 1.

Here n is chosen to match the desired stretching rate such that $n = \frac{d}{v * \Delta t}$.

The chain is considered broken when two neighbouring atoms are more than 4 Å apart.

Even though this looks like we implemented a pull-wait-repeat pulling scheme, we argue that it actually mimics a continuous pulling scheme very closely because of the very small displacement of the end atom. Throughout the simulation the forces on each atom are recorded together with the simulation time. At the end of the simulation we analyse the forces on the terminal atoms to determine the breaking force. This is done by first determining the time the breaking happened, t_{break}, and then doing a linear regression on the forces starting from the initial structure and stopping at t_{break}. The resulting function, $F_{\text{fit}}(t)$, gives us the breaking force $F_b = F_{\text{fit}}(t_{\text{break}})$ and we can compute the uncertainty on that breaking force from the uncertainty of the parameters in the linear fit: $\sigma_F^2 = \sigma_{\text{slope}}^2 t_{\text{break}}^2 + \sigma_{\text{intercept}}^2$. See Supplementary Figure 16.

For $v \leq 10^{-7}$ m/s the chain is effectively in the static limit, because the characteristic time to break thermally is lower than the time between each displacement of the end atom. For $v = 10^{-7}$ m/s it is equal to $n = 10^8$ MD steps between each displacement. $n * \Delta t = 50$ ns is about the longest simulation time we observed.

3.2 Analysis for breaking forces

For each pulling simulation we extract a breaking time, breaking force, and an uncertainty in the breaking force σ_F. This is done 1000 times to allow statistical analysis. For each stretching rate we construct a histogram of breaking forces weighted by $1/\sigma_F^2$. A normal distribution is fitted to this histogram and we determine the most probable breaking force for a given stretching rate as the peak of the distribution and associate an uncertainty from the standard deviation of the distribution.

Comparing the most probable breaking force across stretching rates allows us to see three breaking regimes.

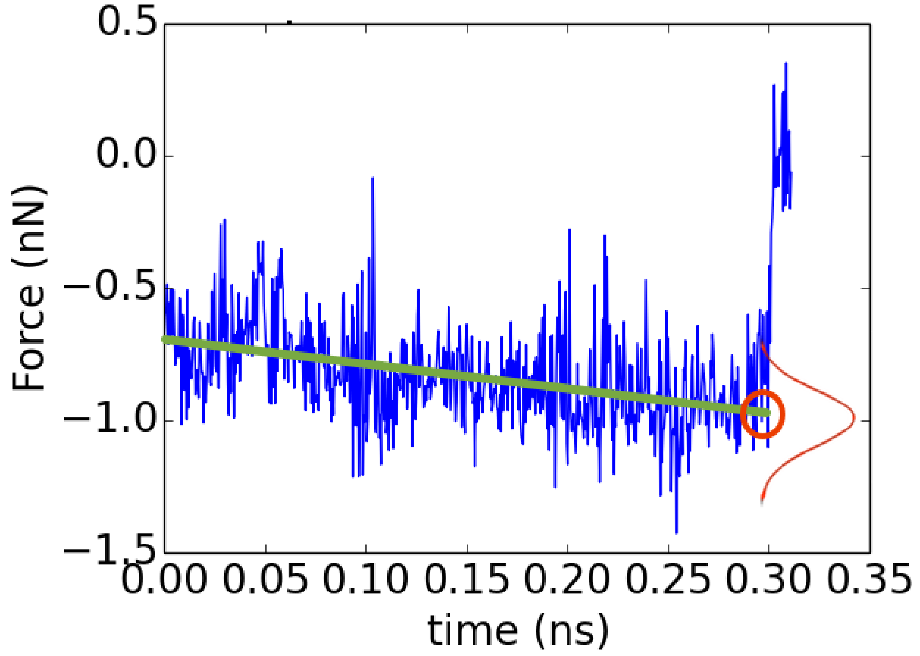

Supplementary Figure 16: Example of a force vs time plot for a single pulling simulation at $v = 0.1\,\mathrm{m/s}$. The forces from the simulation are blue, a straight line fit is shown in green and the fitted value of F_b is shown in red with a normal distribution showing the uncertainty on the breaking force.

3.3 Estimating barrier to break with the nudged elastic band method

To use the rate theory analysis discussed earlier, an estimate of the energy barrier to break is needed (E_a). In addition, we want to know how this energy barrier changes with the stretching distance of the nanowire, to justify our approximation of linear decrease of E_a in eq. (S6). To do this we use the nudged elastic band (NEB) method as implemented in ASE. Given an initial and final structure, NEB estimates the transition state geometry and thereby the energy barrier[18]. We use the same initial structure as were used in all MD simulations. The final state was chosen by sampling several broken structures from the MD simulations and choosing the one that gives the lowest transition state energy. We used EMT as the energy calculator to allow comparisons between NEB results and the MD simulations.

To find length dependence of the energy barrier (α_l), the initial and final structures were modified to increase the end-to-end length of the nanowire, and the NEB calculation was redone. This procedure was repeated 30 times to give a total stretching of 0.06 nm. See Supplementary Figure 17.

3.4 Test for asymmetry in breaking

Because the pulling scheme used is asymmetric (only one end of the nanowire was displaced, while the other end was kept fixed), a test was devised to make sure the mechanical waves introduced by this pulling was sufficiently dampened by the Langevin dynamics to not introduce any asymmetry in the breaking.

Due to the challenges associated with spanning 8 orders of magnitude in pulling speed, there is necessarily a large variation in the number of MD time steps between each displacement. There is a question about how long it takes the wire to equilibrate after one displacement, and how that relates to breaking. If the wire always reaches equilibrium before a new displacement occurs, we can conclude that it is actually breaking from thermal fluctuations in all cases. On the other hand, if we keep displacing the end of the wire before it has reached equilibrium, this will directly lead to breaking. We anticipate that we can distinguish between these two scenarios by looking for asymmetry in the

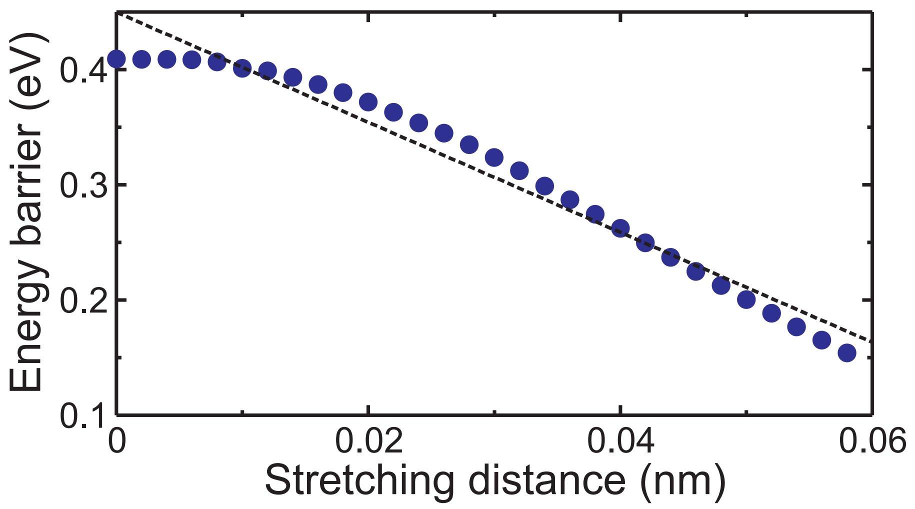

Supplementary Figure 17: The energy barrier to break as a function of the change in end-to-end distance of the gold nanowire. In blue dots, the energy barriers calculated with NEB. The dashed line is a linear fit of the NEB results corresponding to the linearity assumption in eq. (S6).

breaking. In the first case we would expect equal probability for breaking at each end, and for the latter case we expect an overabundance of breaking in the end of the wire being displaced.

We recorded which bond was breaking in each simulation, and saw that it was always the bond between the second and the third atom (breaking left), or the bond between the forth and the fifth atom (breaking right). We hypothesise that this is because the wire will tend to dimerize slightly, weakening the bonds between each dimer pair.

Recording the number of simulations breaking at these bonds for each speed, we plot the ratio of breaking left over breaking right (The breaking symmetry parameter). See Supplementary Figure 18.

Interestingly, the largest deviations from symmetric breaking occur at the lowest pulling speeds when the wire has a very significant chance of breaking before any displacement occurs. Certainly, when we move into the force assisted regime and the terminal atom is frequently displaced, we see no asymmetry in the breaking indicating that this mechanical motion is damped out effectively by the Langevin dynamics. If we move beyond the stretching rates used in this study we can still observe no asymmetry in breaking at $v = 100\,\mathrm{m/s}$, but at $v = 10^3\,\mathrm{m/s}$ the nanowire no longer breaks equally left and right.

For all the stretching rates used, we estimate that just one MD step is needed between each displacement to return the system to equilibrium, because each displacement is very small compared with the thermal motion at room temperature

3.5 Disparity between saturation in maximal force and maximal stretching distance

In the main text Figure 3(a) and 3(b) we see that the maximal force reaches a plateau at $v = 10^{-2}$ m/s but the stretching distance keeps increasing as the stretching rate is increased, for all stretching rates shown. In Supplementary Figure 19 we plot the stretching distance for much higher stretching rates. There we see that we reach a maximal stretching distance at $v = 10^3$ m/s, and we reach the final plateau at $v = 10^4$ m/s.

The final plateau where a distance of 0.16 nm is reached is easily explained. We are pulling at a rate higher than the speed of sound in gold (3240 m/s (CRC)). We are pulling so fast that the other atoms in the wire can't stretch their bonds to compensate, ie. we are just stretching one bond. After the initial relaxation the distance between atoms 5 and 6 is 0.23 nm, and after stretching this single bond for 0.16 nm we reach the breaking condition of 0.4 nm. Thus, the plateau in stretching distance

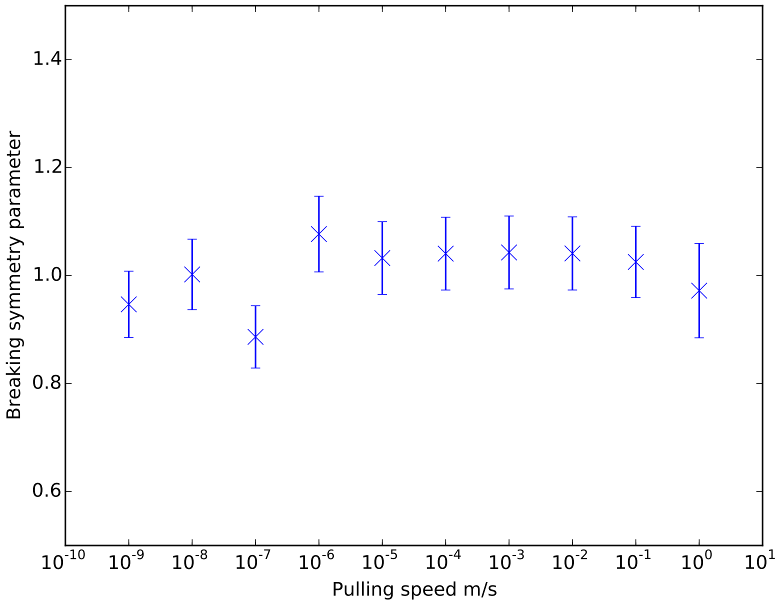

Supplementary Figure 18: Ratio of breaks to the left to breaks to the right. The X marks the most probable symmetry parameter, and the errorbars mark one standard deviation.

for $v > 10^4$ m/s is from stretching just one bond.

It follows that the maximal stretching distance is then reached when the stretching rate is below the speed of sound, so the stretching can be distributed across several bonds. But it should not be at such a low speed that the wire breaks thermally. Thus, the maximal stretching distance can be reached a stretching speed a bit below the speed of sound, $v = 10^3$ m/s.

A question remains: Why do we reach the maximal force at a such a relatively low stretching rate compared to the stretching rate required to reach the maximal stretching distance? From related experiments[19] we expect the stretching length to reach (and be constant at) a maximal value at a much lower stretching rate than what is seen in the simulation. The fact that the breaking forces saturate much earlier than the breaking distances with stretching rate indicate that the system enters an anharmonic regime. In fact a simple simulation where the 6 atom wire is streched in steps and relaxed at each step shows that the forces on the end atoms are anharmonic beyond stretching about 0.3 Å (See Supplementary Figure 20). This of course points to a limitation of the 6 atom model when comparing to experiments. We would not expect room temperature experiments to have such a structure in the junction, we are ignoring motion in the leads which might influence breaking and we are not including any lead surface to stabilize atoms in the junction. Though this is problematic, we limit our discussion to $v < 1$ m/s where we are confident that the simulations are reliable, thereby avoiding the shaky ground of very high rates.

3.6 Most probable stretching distance at cryogenic temperatures

Following the derivations made in Supplementary Note 1.4, we will show the stark contrast between the breaking regimes that are accessible at room temperature and at cryogenic temperatures. We used eqs. S14 and S26 with the following values of the model parameters:

$$\alpha = 0.5\,\text{eV/Å}$$
$$E_a(l_0) = 0.43\,\text{eV}$$
$$\omega = 3 \times 10^{15}\,\text{s}^{-1}$$

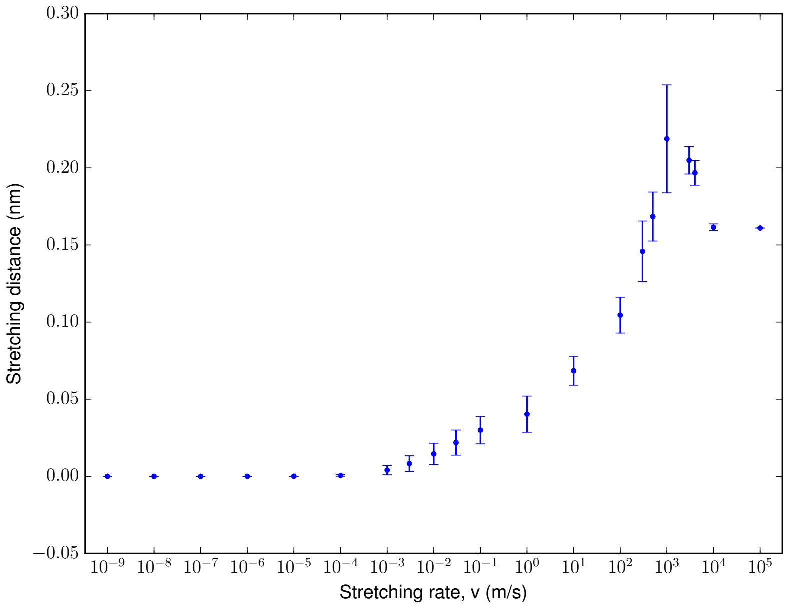

Supplementary Figure 19: Stretching distance for all stretching rates. Symbols are mean values, error bars are standard deviations. The stretching rates $v > 1\,\mathrm{m/s}$ were achieved by lowering the MD timestep and increasing the distance the end atom was moved for each pulling event.

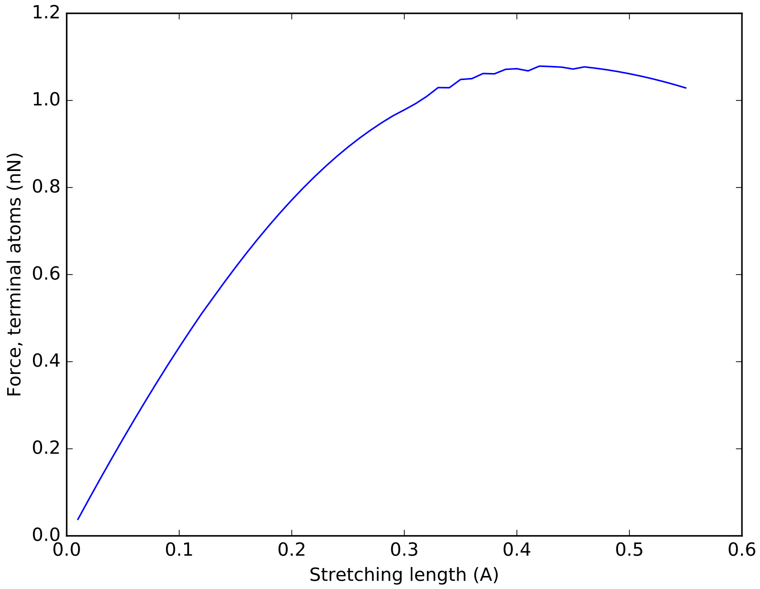

Supplementary Figure 20: Result of a maximal force estimation. This is for the linear 6 atom wire, using EMT to compute the energies.

T (K)	v_c (m/s)	v_{al} (m/s)
4	10^{-540}	10^2
300	10^{-3}	10^4

Supplementary Table 2: For room temperature and cryogenic temperature we calculate the stretching rate where the transition from purely spontaneous breaking to force-assisted breaking takes place, v_c. We also calculate the rate where the transition from force-assisted to activationless takes place, v_{al}.

The initial barrier, $E_a(l_0)$, and the rate with which the barrier decreases with stretching, α_l, was obtained from the NEB calculations described in Supplementary Note 3.3. The attempt frequency, ω, is obtained from fitting survival probability curves for the molecular dynamics simulations at 300 K. These values are the same as are used for making the rate theory predictions shown in the main text, Figure 3.

In Supplementary Table 2 we show the calculated transition stretching rates between the 3 breaking regimes. Note that at 4 K the transition between spontaneous and force-assisted is effectively zero, thus we are practially always in the force-assisted regime. In addition we can say that we are *very* close to the activationless regime, even if the stretching rate is several orders of magnitude lower than the transition rate, v_{al}. This is shown in Supplementary Figure 21, where the most probable breaking length is almost indistinguishable from the activationless limit, over the full range of stretching rates shown. At $T = 4\,\text{K}$ and $v = 10^{-9}\,\text{m/s}$ the most probable breaking distance is 0.84 Å, just 2 pm from the maximal length of 0.86 Å.

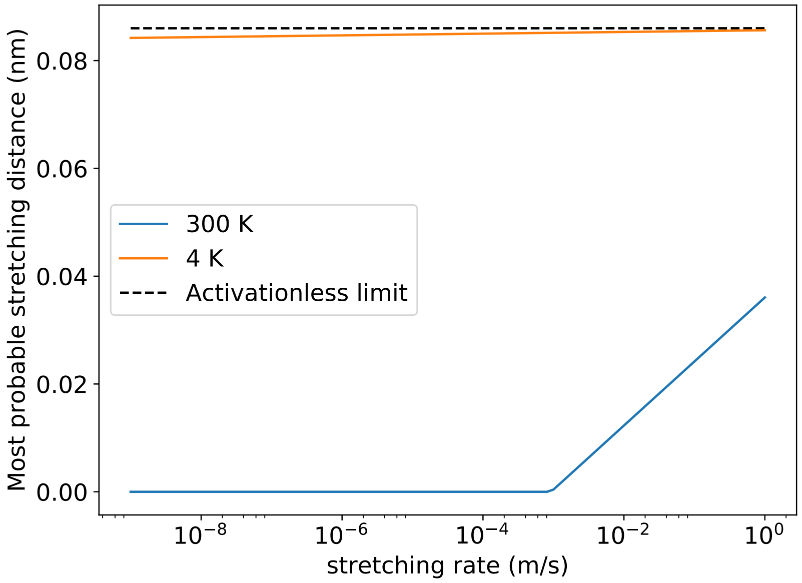

Supplementary Figure 21: The most probable stretching distances for 4 K and 300 K, derived from rate theory. The limit where the barrier to breaking goes to zero (the activationless regime) is marked with a dashed line.

3.7 Simulating point contacts and estimating maximal force

A set of point contact structures was created using ASE and the terminal atoms were constrained. The energy of these structures were minimized with the BFGS method in ASE, then the end-to-end

distance and potential energy was calculated with both EMT and DFT as implemented in GPAW in finite difference mode, with a converged grid-spacing of $h = 0.18$ and with a PBE exchange correlation functional. After each geometry relaxation, the constraints were lifted, the previously constrained atoms at one end of the structure were displaced a short distance and the constraints were applied again. Finally, the energy was minimized again. A potential energy surface can be constructed from the recorded energies and distances. The force is then the gradient of this surface and the maximal force for a point contact is found. A result of this scheme for the 6 atom wire can be seen in Supplementary Figure 20.

Note that the pulling scheme employed here for the point contacts is not the same as the scheme used for the MD simulations. In this case the non-constrained atoms are allowed to fully relax between each displacement of the constrained atoms, whereas the simulations had some fixed number of MD steps to evolve in time between each displacement. This choice eliminates the possibility of breaking thermally for the point contact calculations and therefore the force will reach the maximal value that the bonds can withstand. So the maximal forces reported for point contact breaking must be related to breaking in the activationless regime.

3.8 Transmission for point contacts

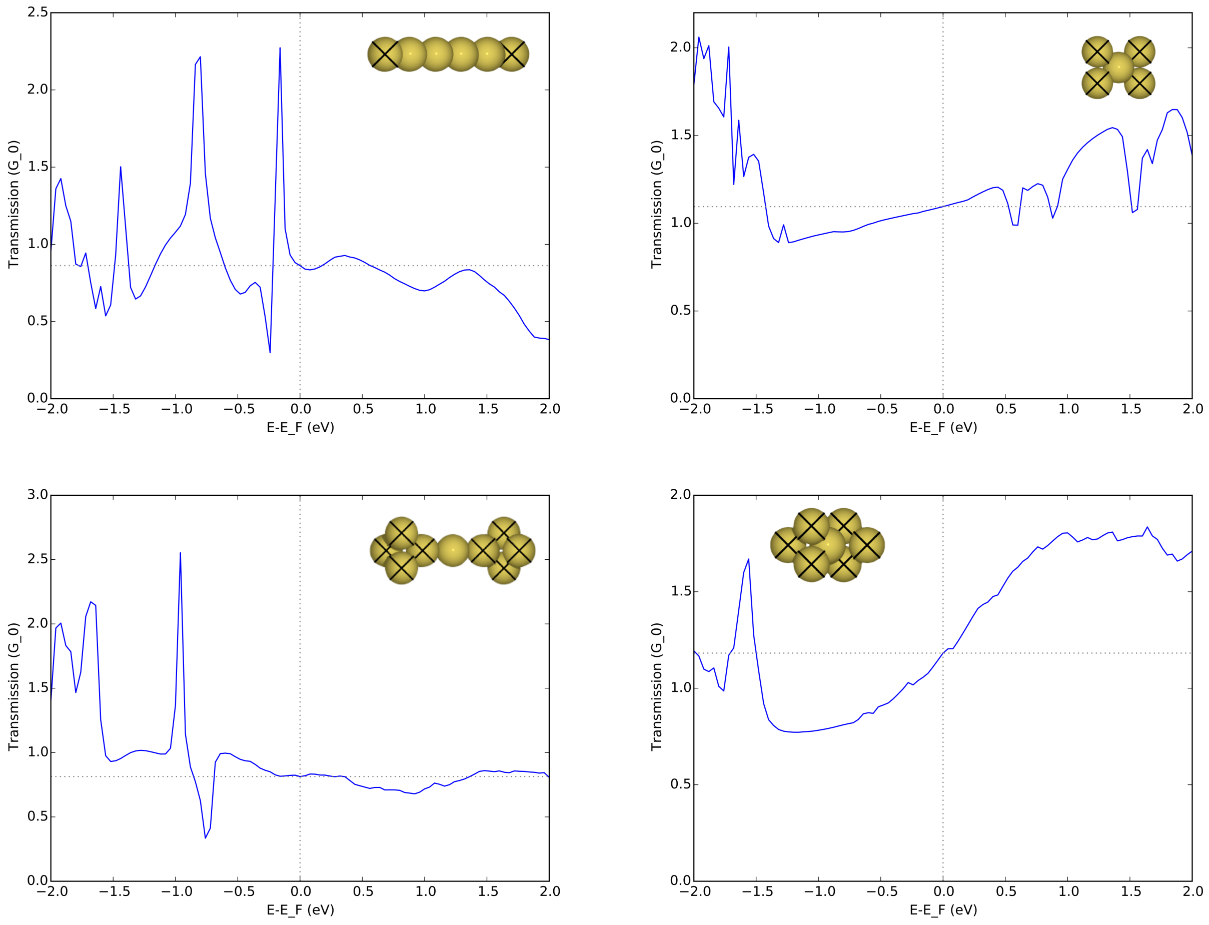

Supplementary Figure 22: Transmission curves for point contact structures. The insets show the structure of the junction between gold leads and correspond to the structures shown in Figure 4(b) of the main text. The value we report for the zero-bias conductance is marked with light grey.

To verify that the point contacts shown in the main text are indeed $1G_0$ we have calculated the transmission of each of them using Atomistix ToolKit version 2014.3[20,21]. The transport calculations

were done with ATK-DFT using GGA exchange correlation, PBE functional with $8 \times 8 \times 100$ k-points and dzp basis set. The transmission spectrum was computed for 101 energies between -2 eV and 2 eV with 8x8 k-point sampling. The transmissions are shown in Supplementary Figure 22.

3.9 Computational details

The simulations and conductance calculations were done on the high performance computing cluster at the Danish Center for Scientific Computing at Copenhagen University. The OS used there is CentOS 6.6. For reference a single simulation of 10^8 MD steps will complete in roughly 5 hours on one E5-2640v3 CPU with 1 GB DDR3-1866 memory.

Supplementary References

[1] Evans, E. & Ritchie, K. Dynamic strength of molecular adhesion bonds. *Biophys. J.* **72**, 1541–1555 (1997).

[2] Barry, D., Parlange, J.-Y. & Li, L. Approximation for the exponential integral (Theis well function). *J. Hydrol.* **227**, 287–291 (2000).

[3] Dudko, O. K., Filippov, A. E., Klafter, J. & Urbakh, M. Beyond the conventional description of dynamic force spectroscopy of adhesion bonds. *Proc. Nat. Acad. Sci. U.S.A.* **100**, 11378–11381 (2003).

[4] Dudko, O. K., Hummer, G. & Szabo, A. Intrinsic rates and activation free energies from single-molecule pulling experiments. *Phys. Rev. Lett.* **96**, 108101 (2006).

[5] Evans, E. Probing the relation between force-lifetime-and chemistry in single molecular bonds. *Annu. Rev. Biophys. Biomol. Struct.* **30**, 105–128 (2001).

[6] Pobelov, I. V. *et al.* An approach to measure electromechanical properties of atomic and molecular junctions. *J. Phys. Condens. Matter* **24**, 164210 (2012).

[7] Rubio, G., Agraït, N. & Vieira, S. Atomic-sized metallic contacts: Mechanical properties and electronic transport. *Phys. Rev. Lett.* **76**, 2302–2305 (1996).

[8] Xu, B., Xiao, X. & Tao, N. J. Measurements of single-molecule electromechanical properties. *J. Am. Chem. Soc.* **125**, 16164–16165 (2003).

[9] Huang, Z., Xu, B., Chen, Y., Di Ventra, M. & Tao, N. Measurement of current-induced local heating in a single molecule junction. *Nano Lett.* **6**, 1240–1244 (2006).

[10] Frei, M., Aradhya, S. V., Koentopp, M., Hybertsen, M. S. & Venkataraman, L. Mechanics and chemistry: single molecule bond rupture forces correlate with molecular backbone structure. *Nano Lett.* **11**, 1518–1523 (2011).

[11] Nef, C., Frederix, P. L. T. M., Brunner, J., Schönenberger, C. & Calame, M. Force–conductance correlation in individual molecular junctions. *Nanotechnology* **23**, 365201 (2012).

[12] Agraït, N., Yeyati, A. L. & van Ruitenbeek, J. M. Quantum properties of atomic-sized conductors. *Phys. Rep.* **377**, 81–279 (2003).

[13] Hong, W. *et al.* An MCBJ case study: The influence of pi-conjugation on the single-molecule conductance at a solid/liquid interface. *Beilstein J. Nanotechnol.* **2**, 699–713 (2011).

[14] Guo, S., Hihath, J. & Tao, N. Breakdown of atomic-sized metallic contacts measured on nanosecond scale. *Nano Lett.* **11**, 927–933 (2011).

[15] Bahn, S. & Jacobsen, K. An object-oriented scripting interface to a legacy electronic structure code. *Computing in Science & Engineering* **4**, 56–66 (2002).

[16] Jacobsen, K., Stoltze, P. & Nørskov, J. A semi-empirical effective medium theory for metals and alloys. *Surf. Sci.* **366**, 394–402 (1996).

[17] Jacobsen, K. W. Bonding in Metallic Systems An Effective Medium Approach. *Comments Cond. Matt. Phys.* **14**, 129–161 (1988).

[18] Henkelman, G., Uberuaga, B. P. & Jónsson, H. A climbing image nudged elastic band method for finding saddle points and minimum energy paths. *J. Chem. Phys.* **113**, 9901–9904 (2000).

[19] Huang, Z., Chen, F., Bennett, P. A. & Tao, N. Single molecule junctions formed via au–thiol contact: Stability and breakdown mechanism. *J. Am. Chem. Soc.* **129**, 13225–13231 (2007).

[20] Brandbyge, M., Mozos, J.-L., Ordejón, P., Taylor, J. & Stokbro, K. Density-functional method for nonequilibrium electron transport. *Phys. Rev. B* **65**, 165401 (2002).

[21] Soler, J. M. *et al.* The siesta method for ab initio order- n materials simulation. *J. Phys. Condens. Matter* **14**, 2745 (2002).
